# Tree species influence soil carbon quality but not total storage across horizons: European beech on Dystric Cambisol and Norway spruce on Entic Podzol

**DOI:** 10.1371/journal.pone.0350656

**Published:** 2026-06-08

**Authors:** Tereza Patrmanová, Andrea Burešová‐Faitová, Václav Tejnecký, Marek Omelka, Ondřej Drábek, Lenka Pavlů, Saven Thai, Jan Kopecký, Markéta Ságová-Marečková

**Affiliations:** 1 Epidemiology and Ecology of Microorganisms, Czech Agrifood Research Center, Drnovská, Prague, Czechia; 2 Department of Genetics and Microbiology, Faculty of Science, Charles University, Viničná, Prague, Czechia; 3 Department of Soil Science and Soil Protection, Faculty of Agrobiology, Food and Natural Resources, Czech University of Life Sciences Prague, Kamýcká, Prague, Czechia; 4 Department of Probability and Mathematical Statistics, Faculty of Mathematics and Physics, Chales University, Sokolovská, Praha, Czechia; 5 Department of Microbiology, Nutrition and Dietetics, Faculty of Agrobiology, Food and Natural Resources, Czech University of Sciences, Kamýcká, Prague, Czechia; Technical University in Zvolen, SLOVAKIA

## Abstract

Tree species influence below-ground soil chemistry and microbial communities, both of which are key drivers of soil formation. The study compared soils under native European beech and first-generation non-native Norway spruce growing at the same site. Soil under beech was classified as Dystric Cambisol, whereas soil under spruce had developed into Entic Podzol. The objective was to link soil chemical processes with microbial community composition and the resulting quantity and quality of soil organic carbon (SOC) across soil horizons. Soil pH and concentrations of available cations and anions were measured together with dissolved organic carbon (DOC), represented by low-molecular-weight organic acids (LMMOA; ion-exchange chromatography). SOC quantity and functional group composition were characterized using Fourier-transform infrared spectroscopy. Microbial abundance and community composition were assessed by 16S/18S rRNA gene amplicon sequencing and droplet digital PCR. Total carbon contents did not differ between soils, but DOC showed horizon-specific differences, with quinate strongly enriched under spruce. More pronounced differences were observed in carbon quality and its vertical distribution. Elevated concentrations and specific forms of Si, Al, P, and S under spruce indicated progressing podzolization, a process absent under beech. Distinct soil conditions and carbon sources supported contrasting microbial communities. Higher pH and labile carbon availability under beech promoted *Pseudomonadota* and *Bacteroidota*, distinguished particularly in the L horizon. In contrast, spruce soils, especially the H horizon were enriched in fungi and metabolically versatile Actinomycetota. Increased abundance of erm resistance genes under spruce also suggested a more competitive microbial environment. Tree species effects on soil properties were detectable throughout the soil profile but weakened with depth. Overall, differences in soil chemistry, microbial communities, and enzymatic activities reflect contrasting decomposition and carbon sequestration pathways, with implications for ecosystem resilience and microbial diversity.

## Introduction

European beech forests are the dominant forest type at elevations between 500 and 1000 m a.s.l. in Central Europe. Over the past two centuries, these climax beech forests have been gradually replaced by Norway spruce to increase timber production, which has significantly altered forest ecosystem dynamics. One major impact has been a decline in soil pH under spruce cover, largely due to the acidic nature of decomposing spruce needles [1]. This acidification was further intensified by atmospheric deposition of acidifying compounds. As a result, spruce vitality has decreased, raising concerns about the influence of spruce forests on carbon cycling, especially in the context of ongoing climate change [2].

Typically, native beech trees uptake cations such as Mg^2+^ and Ca^2+^ from deep soil layers and allow their accumulation in the forest floor forming Dystric Cambisol characterized by slight to moderate weathering of the parent material and lower organic matter content [3,4]. In contrast, the increased soil acidity under spruce contributes to the formation of podzols, often enriched in N and S, while the breakdown of aluminosilicates in an acidic environment leads to release of Al and Fe and their transport with organic matter into deeper soil layers [5–7]. Podzolization process has been described in detail by Sauer et al. [8], for example.

The two soils are further distinguished by microbial communities of decomposers [9], which impact soil organic matter stabilization and carbon sequestration [10]. The size of decomposer communities is proportional to dissolved organic carbon (DOC), thus the better quality litter under beech supports larger microbial communities [10,11]. That has consequences not only for the selection of decomposition pathways but also for the organic matter stability because microbial biomass represents a significant part of recalcitrant carbon in soil [12].

DOC includes a variation of molecules including high molecular mass humic acids and low molecular mass fulvic, phenolic and carboxylic acids, sugars and other small molecules. These molecules are either released during decomposition of larger organic molecules or they are produced newly by microorganisms or plants, while their proportion represents the connections between decomposition pathways and respective microbial decomposers [13,14]. However, uncertainty remains regarding changes in DOC and carbon sequestration during the downward transport from fresh litter to deep soil horizons because both soil processes and microbial communities are rarely studied in a complete vertical soil profile [15,16].

An important but often overlooked component of soil carbon cycling is its link to antibiotic production. Antibiotic-producing microorganisms influence decomposition and carbon sequestration by shaping microbial communities through competitive and cooperative interactions that depend on carbon availability. Antibiotics can inhibit extracellular decomposition enzymes, thereby slowing organic matter breakdown and contributing to carbon sequestration [17–19]. Together with corresponding resistance mechanisms, antibiotic production mediates key microbial interactions and is thus integral to soil decomposition processes [20]. Polyketides are a widespread class of antibiotics synthesized by polyketide synthases (PKSs), while erm genes confer resistance via N-methylation of adenine in 23S rRNA [21]. Because antibiotic activity in forest soils is associated with specific biosynthetic pathways, these genes may serve as indicators linking soil organic carbon (SOC) characteristics to microbial transformation processes [22,23].

Several studies have examined the effects of beech and spruce on soil carbon (C) and nitrogen (N) stocks. Some focused on detailed soil profiles beneath these tree species, providing insights into carbon transformation processes mediated by fungal communities [e.g., 16, 24]. Other studies investigated bacterial communities and their quantitative relationships in beech and spruce forests, but without considering soil formation processes [e.g., 9, 25]. Consequently, our understanding of how soil horizons form through coordinated microbial activity remains limited. To our knowledge, no study has yet jointly addressed the interactions between bacteria and fungi during litter decomposition that lead to the development of distinct soil horizons and soil types.

In this study, we applied a comprehensive methodological approach to characterize entire soil profiles and gain broader insight into soil-forming processes. In addition to measuring total organic and dissolved carbon, we assessed soil organic carbon (SOC) quality by analyzing low-molecular-mass organic acids and functional groups of dominant organic compounds. This approach enabled a closer linkage between organic matter quality and microbial community structure, which was characterized using amplicon sequencing to assess diversity, quantification of bacteria, actinomycetes, and fungi, and measurements of selected hydrolytic enzyme activities involved in decomposition.

To compare these processes under contrasting forest types and to provide insights relevant for forest management—particularly ecosystem resilience related to SOC stabilization and sustained microbial function—we tested the following hypotheses: (1) the two soil types differ in their total carbon stocks; (2) soil chemistry is horizon-specific, reflecting differences in soil-forming processes; (3) microbial community composition differs between forests, particularly in the topsoil where tree litter effects are strongest; (4) antibiotic production and resistance genes are most abundant in the topsoil, reflecting competitive microbial interactions; and (5) the size of microbial communities is reflected in carbon transformation processes along the soil profile.

## Materials and methods

### Site description

This study did not involve human participants and therefore did not require approval from the Czech University of Life Sciences Ethics Committee, nor was informed consent applicable. The research was conducted entirely in the mountain forest environment, and all necessary permissions for fieldwork were obtained from the Nature Conservation Agency of the Czech Republic, in accordance with local and national regulations. The work was done under the permit AOPK/9770/SOPK/2022 PO575/2022.

The study was conducted at one site, where two soil types developed under native beech and the first generation of non-native spruce forests in the Jizera Mountains Beech Forests National Nature Reserve (Czech Republic), a UNESCO World Natural and Cultural Heritage site [26]. The study area is located on the Palicnik hill (Czech Republic), which is formed by uniform granite bedrock. The sampling sites were in two distinct forests (spruce 50.8673 N 15.2544 E; beech 50.8677 N 15.2528 E) situated next to each other on the same bedrock and with the same slope, exposition and precipitation, so a direct comparison of the effect of trees was possible. One forest is composed of native European beech (*Fagus sylvatica* L.), while the other is the first generation of Norway spruce (*Picea abies* [L.] Karst). The ages of the spruce and beech forests are roughly 90 and 170 years, respectively. The prevailing soil types were classified as Entic Podzols under spruce and as Dystric Cambisol under beech forest [27]. The site was previously described, e.g., by Bradová et al. [28].

### Soil sampling

The forest type was not replicated. In each forest, 7 sampling sites were selected, so they had similar conditions to keep the samples as standardized as possible. Sampling sites were chosen to cover the studied areas as uniformly as possible to minimize potential effects of the unobserved factors. The main factors considered were the elevation, which was kept within 10 m, all sites were about three meters from the closest tree. The sites were all on a homogenous substrate with no stones in the profile, and sites where there were rocks coming to the surface were also avoided (S1 Fig in S1 File). The individual sampling sites were located 30–50 m apart; the samples were always collected near a different tree and at a site with unlikely connection by underground water flow from other sites. Soil horizons included: litter (L), fermentation (F) and humified (H) organic horizon, organo-mineral (A) and subsurface B (cambic or spodic) horizon. Seven replicates were taken from all horizons of both spruce and beech forests. In total, 5 horizons in 7 replicates were sampled in each forest. Soil samples were collected from soil pits designated by a steel frame (25 × 25 cm). Organic and organo-mineral A horizons were carefully excavated from the frame and weighed on site. Samples were collected in September 2015. The thickness of each horizon was measured on site from the cleaned soil pit. Steel cylinders of 100 cm^3^ filled with undisturbed soil from each horizon (or carefully collected from several spots in case the horizon was too thin), were used for determination of bulk density of B horizons [29]. For B horizon, a uniform thickness of 25 cm was used. The weight of each horizon per unit area (g.m^−2^) was calculated from its thickness and bulk density [30].

Samples from each horizon were first placed in a large plastic bag, from which subsample for microbial analyses and enzymatic activities was taken into 2 mL Eppendorf tubes, which were immediately placed to a transportable freezer of – 20 °C and upon the arrival to the laboratory to a freezer of −80 °C. Subsamples in small plastic bags were stored at – 20 °C for water extraction and DOC, elemental, organic (low molecular mass organic acids (LMMOA)) and inorganic anions analyses. Part of the soil was dried (40 °C), sieved through 2 mm sieve or sieved and milled for soil organic carbon (SOC) and DRIFT analyses.

### Soil chemical analyses

Soil organic carbon (SOC) content was measured in dry-milled soil samples by a modified Tyurin’s oxidimetric method, using potassium dichromate in sulphuric acid titrated with Mohr’s salt. Fresh samples were subjected to a deionized water extraction (soil/water ratio of 1:10 w/v, 24 h on a reciprocal shaker at a stable laboratory temperature of 20 °C). The suspension was then centrifuged at 4000 rpm for 15 minutes. Finally, extracts were filtered through a 0.45 µm nylon membrane filter (Cronus Membrane Filter Nylon, GB). In aqueous extracts, the following chemical parameters were analyzed: pH, DOC, content of low molecular weight organic acids (LMMOA), inorganic anions and NH_4_^+^ by means of ion chromatography (IC), and content of selected elements using inductively coupled plasma-optical emission spectrometer (ICP-OES). Moisture was determined gravimetrically. The contents of soil parameters were recalculated by dry matter content; pH of aqueous extract was determined potentiometrically (pH meter inoLab pH Level 1, WTW, Germany).

Dissolved organic carbon (DOC) content was determined by a modified wet dichromate oxidation method. Major LMMOAs (quinate, lactate, acetate, propionate, formate, isobutyrate, butyrate, pyruvate, adipate, malate, oxalate and citrate) and inorganic (NO_3_^-^, PO_4_^3-^ and SO_4_^2-^) anions were determined by means of ion-exchange chromatography with suppressed conductivity according to Hubová et al. [30]. The ion chromatograph ICS 1600 (Dionex, Sunnyvale, CA) equipped with IonPac AS11-HC (Dionex) guard and analytical columns was used.

NH_4_^+^ was determined by means of ion-exchange chromatography with suppressed conductivity. The ion chromatograph ICS 90 (Dionex) equipped with IonPac CS16 (Dionex, Sunnyvale, CA) guard and analytical columns was used.

The concentration of selected elements (Al, Ca, Fe, K, Mg, Mn, Na, S and Si) was determined by an iCAP 7000 (Thermo Fisher Scientific, Waltham, MA). Standard reference materials NIST 1643e were used to control the quality of the element determination in the aqueous extract.

Organic matter quality was tested using infrared spectroscopy (DRIFT – diffuse reflectance Fourier transform infrared spectroscopy). The dry soil samples were measured using an infrared spectrometer (Nicolet iS10) and OMNIC 9.2.41 software (Thermo Fisher Scientific). The spectra were recorded at 64 scans in wavenumbers ranging from 4000 to 400 cm^-1^ at a resolution of 4 cm^-1^ and the gold mirror was used as the background of the spectra [31]. The measured reflectance was converted to Kubelka–Munk units (KM). Three indices were calculated from the DRIFT spectra. Potential wettability index (PWI) showed the proportion of relatively hydrophobic (C–H) spectral bands around 2990 and 2854 cm^-1^ to hydrophilic (C = O) functional groups of organic matter in ranges 1700–1740 cm^-1^ and 1600–1640 cm^-1^ [32], i.e., higher PWI values indicated lower soil wettability. Aromaticity index (iAR) was a fraction of aliphatic C–H groups around 2990 and 2854 (ΣAL) from the total of aliphatic (ΣAL) and aromatic C = C groups around 1520 cm^-1^ (AR) cm^-1^: iAR = ΣAL/ (ΣAL + AR) [33]. High values of iAR therefore indicated a lower proportion of aromatic compounds. Decomposition index (iDEC) represented a ratio of carboxylate and aromatic (including lignin) groups in range of 1600–1640 cm^-1^ to polysaccharide spectral bands in range 1100–1125 cm^-1^ [34]. Thus, higher iDEC implied a decline in the intensity of polysaccharide bands, i.e., a depletion of easily utilizable substrates.

### DNA extraction

Soil DNA isolation was performed with method developed by Sagova-Mareckova et al. [35] based on homogenization of soil samples in a bead beater, phenol/chloroform extraction, treatment with CaCl_2_ (1 M CaCl_2_ in 1 M HEPES-NaOH, pH 7) and finally, purification with GeneClean Turbo DNA kit (MP Biomedicals, Irvine, CA).

### Amplicon sequencing and analysis

Fragments of 16S rRNA gene including the variable region V4 were amplified using universal primers with linkers at the 5′-ends CS1_515F (5′-ACACTGACGACATGGTTCTACAGAGTGYCAGCMGCCGCGGTAA-3′) [36] and CS2_806R (5′-TACGGTAGCAGAGACTTGGTCTACGGACTACNVGGGTWTCTAAT-3′) [37]. Reaction was performed in 25 μL volume using GoTaq G2 Hot Start Polymerase (Promega, Madison, WI, USA) and included initialization (95 °C, 300 s); 28 cycles of annealing (55 °C, 45 s) and elongation (72 °C, 30 s), and denaturation (95 °C, 30 s), and final elongation (72 °C, 420 s). Construction of amplicon libraries and sequencing on a MiSeq sequencer (Illumina, San Diego, CA) were done at the Genomics and Microbiome Core Facility, Rush University Medical Center (Chicago, IL).

The primer sequences were removed using a Cutadapt v. 4.9 [38]. The raw sequence data were then processed and analyzed in RStudio v. 2024.04.2 [39] with the R software environment v. 4.3.3 [40] utilizing DADA2 v. 1.28.0 [41] to inspect quality profiles, filter, and trim sequences and then infer amplicon sequence variants (ASVs) and remove chimeras. The resulting amplified sequence variants were classified in Mothur v. 1.47.0 software [42] using the SILVA Small Subunit rRNA Database, release 138.2 adapted for use in Mothur (https://mothur.s3.us-east-2.amazonaws.com/wiki/silva.nr_v138_2.tgz) as the reference database [43]. ASVs of plastids and mitochondria, and those not classified in the domains *Bacteria* and *Archaea* were removed from the ASV table. The sequence table was normalized to a minimum number of sequences per sample and analyzed in Mothur v. 1.47.0 software by tools including Bray-Curtis distance matrices calculation, analysis of molecular variance, (AMOVA), rarefaction and metastats. A total of 2,968,171 16S rRNA gene sequences were mapped into 11,170 prokaryotic ASVs. These ASVs were taxonomically assigned into 31 bacterial and 4 archaeal phyla.

### Quantification of prokaryotes, actinomycetes and fungi

The abundances of total bacteria, actinomycetes (16S rRNA gene), fungi (ITS1 region), type II polyketide synthase (PKS) and *erm* methyltransferase genes were determined by droplet digital PCR (ddPCR) using specific primers (S1 Table in S1 File). Actinomycetes were quantified using primers covering the *Actinomycetota* classes *Actinomycetes* (80.5%), *Acidimicrobiia* (49.2%) and *Rubrobacteria* (6.7%) [44]. Type II PKSs were quantified with primers targeting actinomycete ketoacyl-synthase (KSα), a key enzyme shared by all type II PKSs [45]. Primers for *erm* methyltransferase were designed from gene sequences of GC-rich bacteria [23]. The reaction mixture contained a total volume of 20 µl: 1 × QX200™ ddPCR™ EvaGreen Supermix, 0.25 µM primers and 0.3–200 ng diluted DNA sample. In a QX200™ Droplet Generator (Bio-Rad Laboratories, Hercules, CA), the mixture was partitioned into 20,000 nanoliter-sized droplets. Amplifications were performed in 96-well plates in a C1000 Touch™ Thermal Cycler (Bio-Rad Laboratories). PCR protocol was identical for all the primers, except for the annealing temperature: initial denaturation at 95°C for 5 min, followed by 40 cycles of denaturation at 95 °C for 30 s, annealing at 55 °C (fungi), 56 °C (bacteria, *erm*) or 60 °C (actinomycetes, type II PKS) for 45 s, synthesis at 72 °C for 45 s; and final synthesis at 72 °C for 5 min. Fluorescence intensity was then measured in a Q200™ Droplet Reader (Bio-Rad Laboratories) and data analyzed in a QuantaSoft 1.7.4.0917 (Bio-Rad Laboratories). Based on the fluorescence amplitude, the software assigned droplets to positive or negative based on the presence of target DNA. The number of copies per µl was then calculated to copies per g of soil.

### Hydrolytic enzymes assays

The activities of acid phosphatase (EC 3.1.3.2), alkaline phosphatase (EC 3.1.3.1), β-D-glucosidase (EC 3.2.1.21), cellobiohydrolase (EC 3.2.1.91), chitinase (EC 3.2.1.52), arylsulfatase (EC 3.1.6.1) and lipase (EC 3.1.1.3) were determined using fluorogenic 4-methylumbelliferone substrates, leucine aminopeptidase (EC 3.4.11.1) activity was assessed using a 7-amino-4-methylcoumarin substrate [46]. Activity of alkaline phosphatase was measured in soil and litter samples homogenized in 0.5M Tris buffer (pH 8), whereas all other enzyme activities were assayed in samples homogenized in 50mM acetate buffer (pH 5). Fluorescence was measured using an Infinite 200 PRO Microplate reader (TECAN, Männedorf, Switzerland). Enzyme activities were determined in three analytical replicates and expressed per gram of soil or litter mass.

### Statistical analyses

Because soil horizons represent inherently distinct pedogenic environments, we analyzed horizons separately to capture horizon-specific responses, acknowledging the resulting increase in the number of comparisons. Therefore, only results remaining significant after correction for multiple comparisons are interpreted as statistically robust, whereas uncorrected results are presented to illustrate consistent directional patterns across horizons.

Statistical differences of soil variables and enzymatic activities were assessed by Hotelling‘s two-sample test [47]. The individual sites were considered independent replicates, while the horizons within one site were treated as a multivariate (5-dimensional) response. The test then compares the multivariate means (each component of the mean corresponding to one horizon) between two samples representing spruce and beech forests. Finally the adjustment for multiple comparisons was done by randomly permuting (relabelling) the 5-dimensional response of the individual sites.

Analysis of molecular variance (AMOVA) based on Bray-Curtis distance matrices was used to compare the community composition. The distance matrices were plotted by non-metric multidimensional scaling (NMDS) using Mass package. Environmental fitting using the envfit function in the vegan package showed correlation patterns of linear variables within the ordination space of microbial communities. Only the significantly correlating variables were used in the plots. All other statistical calculations and figures were done in the R software environment v. 4.3.3 [40].

Finally a linear mixed model [48] was used to assess the possible different effect of bacterial and fungal abundance on carbon transformation (e.g., DOC, SOC, enzymatic activities, organic acid concentrations). This model took the carbon transformation as a response. As regressors the abundance and the forest type (spruce and beech) were taken not only separately but also in the interaction. Then the statistical significance of this interaction term proves that the effect of abundance on the carbon transformation in the spruce forest is different from the corresponding effect in the beech forest. Similarly as for the Hotteling’s test the individual site were considered as independent replicates but the horizons within one site were not considered independent. This dependence was modelled by the random effect shared within one site. The adjustment for the multiple comparison was done in the same way as above by randomly permuting (relabelling) the sites.

## Results

### Soil chemical analyses

The most distinct chemical differences between soils under spruce and beech forests were observed for Si, Al, P, S and PO₄³ ⁻ , which remained significantly higher under spruce after correction for multiple testing. Several other soil properties, including pH, Ca, K, Mg and NH₄ ⁺ , showed consistently higher values under beech across horizons, while Fe and NO₃ ⁻ tended to be higher under spruce. However, these differences did not remain statistically significant after correction and are therefore interpreted as directional trends rather than robust effects (S2 and S3 Tables in S1 File).

### Organic matter

Soil organic carbon (SOC) concentrations did not differ between forest types, whereas dissolved organic carbon (DOC) was generally higher under beech, particularly in the L and B horizons.

**Fig 1:**
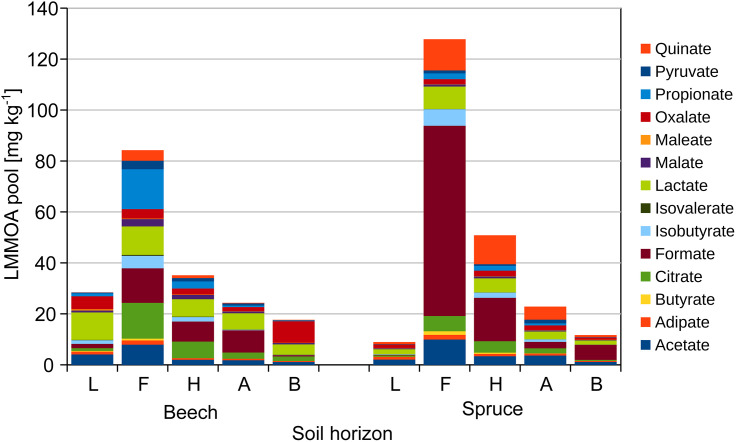
Average amounts of low molecular mass organic acids in the spruce and beech soil horizons (L – litter, F – fermentation, H – humic, A – organo-mineral and B – subsurface) (n = 7).

The total content of all measured low molecular weight organic acids (LMMOA) did not differ under spruce compared with beech (Fig 1, S2 Table in S1 File). Yet, quinate concentrations were significantly higher under spruce and also formate formed a large proportion of LMMOA there (Fig 1, S2 Table in S1 File). Under beech, increased amounts of oxalate and malate were found, but also citrate particularly in L horizon.

DRIFT spectra of soil organic matter generally differed in the two forest soils. which was reflected in systematically higher potential wettability (PWI) and aromaticity indices (ARi) under spruce (S2 Table, S2 Fig in S1 File). In general, PWI representing the ratio between hydrophobic and hydrophilic functional groups, tended to be higher in all horizons under spruce and decreased with depth (S2A Fig in S1 File). Similarly, iAR explaining the relationship between C–H of aliphatic and C = C of aromatic groups had a decreasing tendency towards the B horizons and tended to be higher under spruce (S2B Fig in S1 File). By contrast, the ratio of lignin to carbohydrates estimated by the decomposition index (iDEC) increased in A and B horizons of both stands but did not show any difference between the two forests (S2C Fig in S1 File).

More specifically, the L horizons contained a dominant band of polysaccharides and alcohol or carboxyl groups (1000–1200 cm^-1^) and bands of aliphatic C–H groups around 2990 and 2854 cm^-1^. In the F horizons, there was a relative increase (relative to the polysaccharide band) of the C = O groups of ketones and amides with C = C aromatic rings (around 1650 cm^-1^). This trend continued towards the H horizon but distinct bands of aliphatic components of organic matter, or bands of carboxyl groups were still present (1720–1730 cm^-1^). More intense bands of mineral soil components began to appear towards B horizon compared to the A horizon. The difference between spruce and beech stands was in higher relative proportion (compared to the band around 1650 cm^-1^) of carboxyl groups in all surface organic horizons of the spruce stand (S3 Fig in S1 File).

### Microbial community

**Fig 2:**
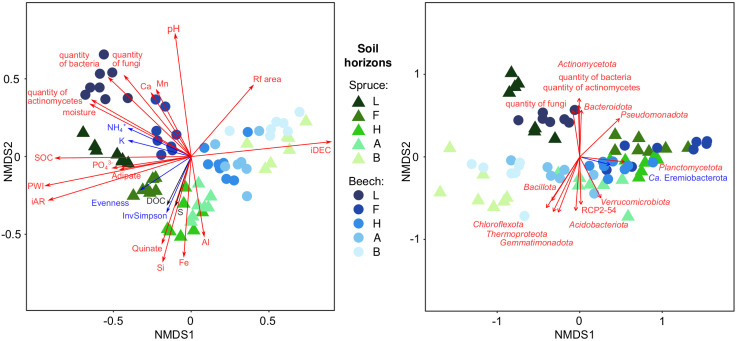
Differences between microbial communities (A) and chemical characteristics (B) of the beech (blue circles) and spruce (green triangle) samples. Non-metric multidimensional scaling was based on matrix of Bray-Curtis distances. Linear vectors represent chemical variables and quantities significantly correlating with the structure of bacterial community or chemical properties A, or relative abundancies of bacterial phyla B (p < 0.001 red vectors; 0.001 < p < 0.01 blue vectors; 0.01 < p < 0.05 black vectors).

Non-metric multidimensional scaling (NMDS) revealed a clear separation of microbial communities between spruce and beech stands, most pronounced in the L and F horizons (Fig 2A, S4 Table in S1 File). With increasing depth, communities in the H, A and B horizons became more similar to each other. Environmental fitting indicated that variation in microbial community composition in beech L and F horizons was closely associated with total bacterial, actinomycete and fungal abundance, as well as with moisture, Ca and Mn concentrations (all *p* < 0.001), and lactate (*p* < 0.05). In contrast, communities in the upper spruce soil horizons aligned strongly with SOC, PO₄³ ⁻ , adipate content, potential wettability index (PWI), and index of aromaticity (iAR) (all *p* < 0.001). Spruce H and A horizon communities further correlated with Si, Al, Fe and quinate (*p* < 0.001), as well as DOC and S (*p* < 0.05). The decomposition index (iDEC) was consistently associated with B-horizon communities of both forest types (Fig 2A).

A NMDS based on soil chemical properties showed weaker separation between spruce and beech soils than that observed for microbial community composition (Fig 2B), although L horizons remained relatively distinct. Separation along the L-horizon gradient was associated with actinomycete and fungal abundance (*p* < 0.001) and with the relative abundances of *Bacteroidota* and *Actinomycetota*. With increasing depth, F and H horizon samples aligned with higher relative abundances of *Pseudomonadota* and *Planctomycetota* (*p* < 0.001) and *Candidatus* Eremiobacterota (*p* < 0.01). Samples from A horizons of both forests were associated with *Verrucomicrobiota*, RCP2–54, *Acidobacteriota*, *Gemmatimonadota*, *Chloroflexota*, *Bacillota* and *Thermoproteota* (*p* < 0.001).

### Microbial communities

**Fig 3:**
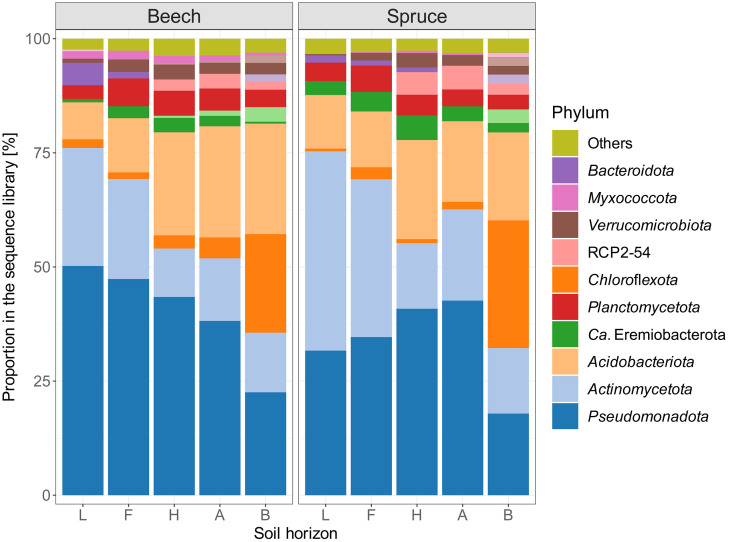
Proportions of bacterial and archaeal phyla in the sequence library of soil horizons (L – litter, F – fermentation, H – humified, A – organo-mineral and B – subsurface horizon) under beech and spruce (n = 7).

Microbial communities consisting of bacteria and archaea differed significantly between both forests in all horizons (AMOVA, p < 0.001; S5 Table in S1 File). Between spruce and beech soil communities, 2,142 ASVs differed significantly (Metastats, p < 0.05), out of which 1,309 ASVs increased under the beech and 833 ASVs under the spruce canopy. Based on the assignment of ASVs separating two forests into corresponding taxonomic units, the discriminant ASVs of *Pseudomonadota*, *Acidobacteriota*, *Myxococcota* and *Bacteroidota* were proportionally higher in the beech forest, while *Actinomycetota*, *Candidatus* Eremiobacterota and RCP2–54 prevailed under spruce (Fig 3, S4 Fig in S1 File). Among the ASVs increased in the beech stand, those of *Actinomycetota*, *Armatimonadota* and *Bacteroidota* were enriched in L horizon compared to the lower ones, while ASVs of *Thermoproteota*, *Dependentiae*, *Desulfobacterota*, *Elusimicrobiota*, *Nitrospirota*, *Patescibacteria*, RCP2–54 and *Thermoplasmatota* were reduced in the top layer (S4A and S4B Figs in S1 File).

The proportion of bacterial phyla differed between the two forests (Fig 3). In the beech stand, the phylum *Pseudomonadota* was dominant (51.1% in the sequence library) in the L horizon, but the proportion decreased with depth. Conversely, the proportion of *Pseudomonadota* tended to increase in deeper horizons of spruce, except for the B horizon where it was the lowest (18.4%). The predominant phylum in upper layers in both stands was *Actinomycetota*. A trend of growing proportions of *Acidobacteriota* and *Chloroflexota* with depth was apparent in both stands. Overall, the most substantial differences between the forests were in the soil’s upper layers and the least prominent in the B horizon. More specifically, in beech, L horizons were enriched by *Sphigomonadaceae*, *Commanodaceae Microbacteriaceae*, *Actinospicaceae*, *Frankiaceae*, *Streptomycetaceae* and *Micromonosporaceae*, while in spruce by *Acetobacteraceae*, *Solirubrobacteraceae, Mycobacteraceae*, *Acidothermaceae*. Starting with H horizons, the communities were becoming more similar (Fig 2, S5 and S6 Figs in S1 File).

The diversity of microbial communities assessed by inverse Simpson index was generally higher under beech (S2 Table in S1 File). Rarefaction curves confirmed the increased microbial diversity in the beech stand with the largest difference in horizon H and lowest in horizon B (S7 Fig in S1 File).

In comparison with the individual horizons between beech and spruce forests (S5 and S6 Figs, S8 Fig in S1 File), horizon L was mostly enriched in *Pseudomonadota*, (*Xanthobacteraceae*, *Sphingomonadaceae*, *Caulobacteraceae*, *Comamonadaceae*) and *Actinomycetota* (*Frankiaceae*, *Microbacteriaceae*, *Micromonosporaceae*) under beech, while *Actinomycetota*, particularly *Solirubrobacteraceae*, *Acidothermus*, *Mycobacterium* and *Acidimicrobiaceae*, and *Pseudomonadota* (*Acetobacteraceae*, *Xanthobacteraceae*, *Caulobacteraceae*) were enriched under spruce. The communities of horizon F followed a similar pattern as in L horizon. In horizon H, the majority of the differentiating taxa were from *Pseudomonadota*, from the same families as in F horizon with the addition of *Elsterales* under beech. In horizons A and B the differences were less pronounced (S8 Fig in S1 File).

### Microbial abundances and secondary metabolites

**Fig 4:**
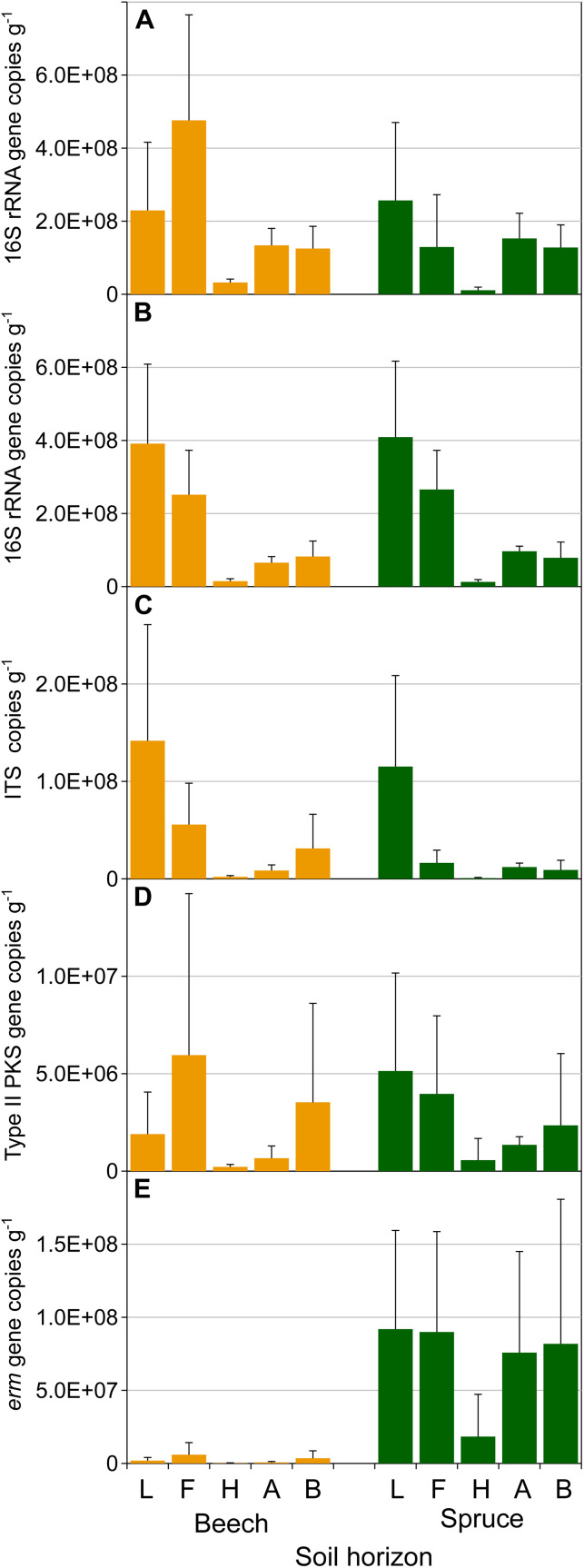
Quantities of total bacteria (A), actinomycetes (B), fungi (C), type II PKS (D) and *erm* methyltransferase genes (E) in soil horizons (L – litter, F – fermentation, H – humified, A – organo-mineral and B – subsurface) under beech (blue) and spruce (green) determined by droplet digital PCR (n = 7).

In both forests abundances of microorganisms as well as genes related to secondary metabolism were reduced in H horizon (Fig 4). Abundances of bacteria and actinomycetes tended to be higher under beech particularly in L and F horizons. Abundances of PKSII genes did not differ between the two forests, however, *erm* resistance tended to be higher under spruce (Fig 4).

### Activities of extracellular hydrolytic enzymes

Enzymatic activities generally decreased with depth in both soils. The most differentiating enzyme between beech and spruce was alkaline phosphatase, which tended to have higher activity under beech in the whole soil profile. The other enzymatic activities, i.e., acid phosphatase, cellobiohydrolase and arylsulfatase did not differ between the forests (S9 Fig, S2 Table in S1 File).

### The size of microbial communities and carbon transformation

Mixed linear models showed that several relationships in carbon processing tend to differ between beech and spruce soil profiles. Suggestive differences between the two soils were determined for relationship of bacterial abundance and leucine-aminopeptidase, DOC, lipase, formate, adipate, cellobiohydrolase, SOC, iAR, iDEC and thickness of horizons, while fungal abundance was related to lactate, formate, DOC and oxalate (S6 Table in S1 File). Significant relationships were found between bacterial abundance and leucine-aminopeptidase, DOC and fungal abundance and lactate. In all those situations, there was a positive slope for beech and neutral or negative for spruce (S10 Fig in S1 File).

## Discussion

The study was conducted at a single site with two adjacent forest stands, allowing a direct comparison under nearly identical environmental conditions. This design likely reduced confounding factors and has been used in previous studies investigating tree-species effects on soil processes [16,24,49]. The two forest types were primarily distinguished by soil chemical properties, most notably higher concentrations of Si, Al, P, and S under spruce, which were subsequently associated with differences in microbial community composition and activity.

The observed chemical differences are are consistent with incipient podzolization under spruce, a process not observed under beech [50]. This interpretation is consistent with earlier findings showing that coniferous stands tend to enhance podzolization intensity [51,52]. Although podzolization is a long-term soil-forming process requiring centuries for full profile differentiation, vegetation-induced chemical changes can occur on much shorter timescales, and increased podzolization following conifer establishment has been repeatedly documented [51,52].

In contrast, cambisols developed under beech are typically characterized by lower organic matter contents and limited vertical transport of iron and aluminium [3,4]. The contrasting soil properties under spruce and beech can be further explained by differences in base cation inputs via litter. Beech litter is enriched in K ⁺ , Ca² ⁺ , and Mg² ⁺ , whereas soils under spruce are depleted in these cations, resulting in lower pH and enhanced mobilization of Al, Fe, and Si, which are subsequently translocated to deeper horizons [7]. This base-cation enrichment under beech is typically associated with higher humus quality, faster mineralization, and higher turnover rates [3,50]. Nevertheless, such patterns are not universal, as illustrated by studies from northern Czech mountain forests where mineralization rate did not differ between beech and spruce stands [53].

Despite these contrasting pedogenic developments, total carbon stocks did not differ between beech and spruce soils, contrary to our hypothesis and several earlier studies reporting higher carbon stocks under spruce [25,53–55]. Our results align with those of Rensedokken et al. [16], who also observed marked differences in vertical carbon distribution without differences in total soil carbon stocks, suggesting that tree species effects may preferentially influence carbon allocation rather than overall storage.

In our study, bacterial abundance tended to be higher under beech stands, particularly in the topsoil, which is consistent with the study by Horvat et al. [25]. That is parallel to the increased DOC concentrations observed in the two upper horizons of the beech forest and supports the previously described positive relationship between bacterial biomass and DOC as a readily available carbon source [e.g., 56].

These findings draw attention to differences in the composition of low-molecular-mass organic acids (LMMOA), which constitute only a small fraction of total DOC but substantially influence the acidity of the DOC solution [13,57]. In our study, LMMOA accounted for 39% of DOC under beech compared to 26% under spruce, further supporting the interpretation of a greater neutralization capacity of beech stands through mobilized base cations, as reflected by the higher soil pH under beech [7]. LMMOA are predominantly derived from plant material in the upper soil horizons [15,58] and may therefore contribute to the early divergence of soil processes that are later observed also in deeper horizons. This is further reflected in higher wettability and aromaticity indices under spruce, suggesting a greater proportion of non-polar carbohydrates, likely originating from needles, which are rich in waxes and terpenes and are consequently more resistant to decomposition [59,60].

Further to that, in fermentation horizons (F) characterized by partially decomposed plant residues and enhanced DOC levels are due to both leaching from litter horizon and production by microbial decomposers [61]. The differences in the decomposition rate seem even amplified under beech in F horizon, because only propionate and malate remained higher in beech, while a significant increase of quinate was observed under spruce. That suggests higher turnover in spruce and may indicate an active microbial community [62].

In lower horizons, both soil chemistry and microbial communities overlapped between the two forests, showing the unifying influence of bedrock.

Microbial communities followed soil chemistry similarly to other studies [62,63]. Top horizons under beech were characterized by increased proportions of *Pseudomonadota* and *Bacteroidota* possibly because they generally prefer neutral soils and readily decomposable organic matter [64,65]. In contrast, *Actinomycetota* tended to be higher under spruce, which together with some of their enriched families indicated that organic matter might be difficult to decompose. These bacteria are often specialized in decomposition of compounds inaccessible to other microorganisms [66] and were described as late-stage substrate generalists capable of decomposing sequestrated carbon [67]. *Acidimicrobiaceae* were probably enriched under spruce due to lower pH which parallels results by Choma et al. [9], who also found these taxa setting apart beech and spruce stands.

More specifically under beech, particularly *Xanthobacteraceae* and *Caulobacteraceae* were likely stimulated by available organic C, and elevated *Caulobacteraceae* might also be related to available N. Those two families form putative keystone associations in decomposing bacterial networks, which corresponds to our observations because they are always enriched together [68]. Further to that, nitrogen enrichment under spruce might be reflected in the dominance of *Conexibacter*, which can reduce nitrate to nitrite and may indicate intensified nitrogen cycling [69]. Finally, high frequency of mycobacteria under spruce specifically corresponds to the recalcitrant needle litter, because that group is known to decompose complex organic compounds [70].

Specialized groups of *Actinomycetota* were significantly elevated in lower horizons under spruce, particularly *Acidothermaceae* and *Solirubrobacteraceae*. The genus *Acidothermus* thrives in acidic soils and decomposes cellulose and hemicellulose [9,71], while *Solirubrobacterales* are known producers of secondary metabolites [72]. By the time the organic substrate reaches the subsurface horizons, readily available nutrients are mostly utilized, and the character of remaining organic matter becomes dominated by alkyl groups [73]. This might support a diverse range of oligotrophic taxa, such as *Chloroflexota* and *Bacillota* [62]. In accordance, among the discriminant taxa of the two forests were those corresponding to *Chloroflexota*, *Bacillota,* and also *Nitrospirota*.

Higher numbers of *erm* resistance genes were accordingly observed in upper horizons of beech and lower horizons of spruce. Their distribution may suggest competitive relationships forced by antagonistic mechanisms [18]. As a consequence, the decomposer community in those horizons may be specializing by the litter source leading to community divergence [15,17]. Possibly, the harsh conditions of low-quality carbon and low pH select only for smaller but highly specialized community [74]. Differences between the soils predicted by relationships between fungal abundance and lactate, bacterial abundance and DOC, and leucine aminopeptidase activity may further suggest that microbial abundance under beech was indeed related to the carbon transformation processes but under spruce that was not the case because under beech the relationship was positive, while under spruce it was neutral or negative [9,75].

Finally, fungal biomass did not differ between the two forest types, suggesting that fungi may act as site generalists [67]. This contrasts with the findings of Uroz et al. [49], who showed that fungal communities were associated with tree litter, while bacterial communities showed no such specificity.

## Conclusions

At the study site of Jizera Mountains, where native European beech and the first generation Norway spruce grow next to each other and two different soil types developed, the total stocks of soil organic carbon (SOC), dissolved organic carbon (DOC), and overall microbial biomass did not differ between forests. However, carbon distribution across soil horizons, DOC and SOC quality were soil- and horizon-specific and linked to soil forming processes particularly podzolization under spruce. Similarly, microbial community composition was closely linked to carbon quality inputs, with the most pronounced differences occurring in the litter horizons and least in mineral horizon suggesting that depth reduces tree-species effects.

The soil under spruce was enriched in Si, Al, P and S reflecting the podzolization process. LMMOA was dominated by quinate, and microbial community was characterized by *Actinomycetota*, particularly groups known for decomposing complex organic compounds.

In comparison, the soil under beech tended to have higher pH, microbial diversity, better quality of SOC, lower hydrophobicity and aromaticity compared to spruce forests.

The differences in soil processes induced by different trees showed that soil microbial community of beech forest was quantitatively related to decomposition processes at that site, while in spruce forest was not. That may have implications for carbon turnover and buffering of soil pH, which is essential for maintaining functionally diverse microbial communities. Reduced wettability under spruce further suggests potential limitations in water retention capacity, a critical factor as forests face increasingly frequent extreme rainfall events under climate change. Moreover, soil acidification beneath spruce forests not only constrains tree performance but progressively reduces decomposer diversity, as most microorganisms and soil fauna are adapted to near-neutral pH conditions.

Nonetheless, these findings are derived from a single study site, which limits their broader generalization. Further research across a wider range of sites is therefore needed to more robustly quantify the links between microbial community structure and carbon fluxes, thereby improving predictive capacity and informing management recommendations.

## Supporting information

S1 File S1-S6 Tables; S1 – S10 Figs. (PDF)

S1 Data Raw data of all measurements. (XLSX)
