# Supplementary material for: Tree species influence soil carbon quality but not total storage across horizons: European beech on Dystric Cambisol and Norway spruce on Entic Podzol
Source: PLoS One. 2026 Jun 8;21(6):e0350656. doi: 10.1371/journal.pone.0350656 (PMC13245778; doi:10.1371/journal.pone.0350656)
Supplement: S1 File — (PDF) [file pone.0350656.s001.pdf]

Supplementary Information for

Tree species influence soil carbon quality but not total storage across horizons: European beech on Dystric Cambisol and Norway spruce on Entic Podzol

Tereza Patrmanová[a,b], Andrea Burešová-Faitová[a], Václav Tejnecký[c], Marek Omelka[d], Ondřej Drábek[c], Lenka Pavlů[c], Saven Thai[c], Jan Kopecký[a], Markéta Ságová-Marečková[a,e,*]

[a] Epidemiology and Ecology of Microorganisms, Czech Agrifood Research Center, Drnovská 507, 161 00 Prague 6, Czechia

[b] Department of Genetics and Microbiology, Faculty of Science, Charles University, Viničná 5, 128 44 Prague 2, Czechia

[c] Department of Soil Science and Soil Protection, Faculty of Agrobiology, Food and Natural Resources, Czech University of Life Sciences Prague, Kamýcká 129, 165 00 Prague 6, Czechia

[d] Department of Probability and Mathematical Statistics, Faculty of Mathematics and Physics, Chales University, Sokolovská 49/83, Praha 8, Czechia

[e] Department of Microbiology, Nutrition and Dietetics, Faculty of Agrobiology, Food and Natural Resources, Czech University of Sciences, Kamýcká 129, 165 00 Prague 6, Czechia

[*] Corresponding author. E-mail address: mareckova@af.czu.cz

This PDF file includes:

Tables S1 to S6

Figures S1 to S10

S1 Table. Primers used for quantification by digital droplet PCR.

Primer	Sequence (5'-3')	Sense	Target	Reference
16Seu27f	AGAGTTTGATCMTGGCKCAG	forward	16S rRNA (*Bacteria*)	Čermák et al. (2008)
783r-a	CTACCAGGGTATCTAATCCTG	reverse	16S rRNA (*Bacteria*)	Sakai et al. (2004)
783r-b	CTACCGGGGTATCTAATCCCG	reverse	16S rRNA (*Bacteria*)	Sakai et al. (2004)
783r-c	TACCCGGGTATCTAATCCGG	reverse	16S rRNA (*Bacteria*)	Sakai et al. (2004)
Acti-1154	GRDACYGCCGGGGTYAACT	forward	16S rRNA (*Actinomycetes*)	Pfeiffer et al. (2014)
Act-1339	TCWGCGATTACTAGCGAC	reverse	16S rRNA (*Actinomycetes*)	Pfeiffer et al. (2014)
ITS1F	CTTGGTCATTTAGAGGAAGTAA	forward	ITS1	(Buée et al., 2009)
ITS2	GCTGCGTTCTTCATCGATGC	reverse	ITS1	(White et al., 2009)
ks2f	GGITGCACSTCIGGIMTSGAC	forward	KSα (type II PKS)	Wawrik et al. (2005)
ks2r	CCGATSGCICCSAGIGAGTG	reverse	KSα (type II PKS)	Wawrik et al. (2005)
rB1f	ARCWCGGYCAGAAYTTYCT	forward	*erm*	Čermák et al. (2008)
rB1r	CGSGCSACYTCCCAYTG	reverse	*erm*	Čermák et al. (2008)

S2 Table. Differences between beech and spruce forest soils across all horizons. Data are calculated per soil dry weight where applicable, and represented as means ± standard deviations (n = 7-8). Hotteling's two sample test is used to compare the beech and spruce stands. Permutation of sites is used to adjust the p-values for the multiple comparison problem.

Soil characteristics	Stand	Soil horizon					**T2 stat.**	**P**	**P_{adj}**
		**L**	**F**	**H**	**A**	**B**			
Si [mg kg⁻¹]	beech	0.69 ± 0.62	5.15 ± 3.50	7.31 ± 6.04	2.72 ± 2.93	0.62 ± 0.81	32.03	<0.001	0.003
	spruce	8.42 ± 3.81	15.3 ± 4.3	14.8 ± 3.4	8.54 ± 5.10	6.92 ± 2.01			
Al [mg kg⁻¹]	beech	0.55 ± 0.46	8.61 ± 5.02	14.8 ± 5.6	5.84 ± 3.29	0.82 ± 0.71	29.7	<0.001	0.004
	spruce	2.30 ± 1.28	5.53 ± 2.25	12.1 ± 1.8	8.60 ± 1.96	4.95 ± 1.66			
Quinate [mg kg⁻¹]	beech	0.07 ± 0.06	4.13 ± 6.50	1.10 ± 0.65	0.22 ± 0.12	0.20 ± 0.16	21.93	<0.001	0.005
	spruce	0.87 ± 1.57	12.3 ± 8.2	11.4 ± 4.6	5.10 ± 3.93	0.78 ± 0.9			
P [mg kg⁻¹]	beech	9.3 ± 3.83	23.4 ± 13.6	4.86 ± 2.19	1.39 ± 0.95	0.11 ± 0.16	20.33	<0.001	0.007
	spruce	11.4 ± 2.6	27.7 ± 3.7	12.9 ± 2.8	6.06 ± 1.79	2.15 ± 0.29			
SO_4^{2-} [mg kg⁻¹]	beech	13.5 ± 4.6	53.8 ± 28.7	27.6 ± 6.6	15.0 ± 5.7	21.4 ± 5.2	15.59	0.001	0.023
	spruce	21.4 ± 27.4	39.2 ± 14.4	41.5 ± 15.1	38.5 ± 13.5	35.2 ± 24.0			
PO_4^{3-} [mg kg⁻¹]	beech	24.0 ± 10.2	63.4 ± 45.4	3.36 ± 1.57	1.03 ± 0.82	0.39 ± 0.16	14.97	0.001	0.025
	spruce	23.4 ± 6.0	70.9 ± 17.5	14.6 ± 6.1	3.17 ± 1.79	0.18 ± 0.11			
Quantity *erm* genes [gene copies g⁻¹]	beech	1.90E+06 ± 2.16E+06	5.95E+06 ± 8.29E+06	2.19E+05 ± 1.25E+05	6.60E+05 ± 6.30E+05	3.53E+06 ± 4.64E+06	15.3	0.002	0.083
	spruce	9.19E+07 ± 6.75E+07	8.99E+07 ± 6.88E+07	1.83E+07 ± 2.90E+07	7.58E+07 ± 6.92E+07	8.18E+07 ± 9.03E+07			
alkaline phosphatase [nmol min⁻¹ g⁻¹]	beech	43.05 ± 13.81	42.77 ± 17.59	16.09 ± 3.58	9.280 ± 5.349	7.405 ± 2.550	8.38	0.005	0.146
	spruce	17.99 ± 7.94	15.73 ± 6.36	7.109 ± 2.664	3.450 ± 1.053	3.475 ± 1.876			
Ca [mg kg⁻¹]	beech	11.2 ± 7.32	17.7 ± 15.1	3.50 ± 1.74	1.44 ± 0.72	0.90 ± 0.42	7.33	0.007	0.211
	spruce	1.08 ± 0.48	5.61 ± 1.06	1.66 ± 0.53	0.79 ± 0.40	0.81 ± 0.36			
Citrate [mg kg⁻¹]	beech	1.09 ± 1.39	14.0 ± 15.8	6.49 ± 2.95	2.45 ± 1.14	1.78 ± 1.26	7.17	0.008	0.221
	spruce	0.52 ± 0.80	5.99 ± 6.48	4.47 ± 1.91	1.95 ± 1.10	0.36 ± 0.24			
chitinase [nmol min⁻¹ g⁻¹]	beech	48.78 ± 30.98	35.64 ± 19.54	4.693 ± 3.32	2.086 ± 1.524	1.608 ± 0.618	6.98	0.009	0.229
	spruce	66.17 ± 36.99	15.37 ± 8.67	4.245 ± 2.040	1.916 ± 0.623	1.043 ± 0.794			
K [mg kg⁻¹]	beech	46.4 ± 16.8	110 ± 42	48.2 ± 8.3	17.0 ± 6.9	6.86 ± 3.94	6.9	0.009	0.235
	spruce	19.3 ± 3.8	54.1 ± 7.4	40.4 ± 11.8	15.2 ± 3.8	2.74 ± 1.12			
NO_3^- [mg kg⁻¹]	beech	5.91 ± 5.92	29.1 ± 37.5	37.6 ± 33.6	16.3 ± 7.1	6.37 ± 3.56	6.47	0.011	0.278
	spruce	16.7 ± 5.9	33.0 ± 14.5	16.5 ± 21.5	21.1 ± 49.0	4.24 ± 3.47			
Fe [mg kg⁻¹]	beech	0.15 ± 0	1.77 ± 2.70	3.79 ± 3.87	1.35 ± 1.93	0.11 ± 0.01	6.38	0.011	0.288
	spruce	0.14 ± 0.01	2.60 ± 2.10	8.62 ± 4.57	10.2 ± 2.42	0.54 ± 0.95			
Inverse Simpson index	beech	127 ± 37	116 ± 28	144 ± 20	142 ± 38	156 ± 36	6.05	0.013	0.333
	spruce	102 ± 31	126 ± 23	93.9 ± 29.1	88.5 ± 22.8	115 ± 40			
PWI	beech	0.23 ± 0.02	0.17 ± 0.01	0.13 ± 0.02	0.10 ± 0.01	0.07 ± 0.02	5.93	0.014	0.349
	spruce	0.27 ± 0.02	0.24 ± 0.04	0.18 ± 0.03	0.13 ± 0.03	0.07 ± 0.02			

DOC [mg kg^{-1}]	beech	69.4 ± 21.8	678 ± 358	306 ± 115	144 ± 59	57.8 ± 17.4	6.43	0.015	0.375
	spruce	34.4 ± 38.4	474 ± 251	461 ± 132	218 ± 73	27.3 ± 15.4			
Mg [mg kg^{-1}]	beech	1.56 ± 1.02	2.70 ± 1.74	1.86 ± 0.53	1.07 ± 0.21	0.80 ± 0.22	5.73	0.015	0.383
	spruce	0.28 ± 0.13	1.63 ± 0.30	1.30 ± 0.20	0.93 ± 0.20	1.04 ± 0.43			
pH (H_2O)	beech	4.99 ± 0.21	4.20 ± 0.29	4.15 ± 0.22	4.32 ± 0.19	4.51 ± 0.10	5.46	0.018	0.434
	spruce	4.59 ± 0.12	3.90 ± 0.43	3.79 ± 0.11	3.93 ± 0.08	4.32 ± 0.09			
Quantity of bacteria	beech	2.29E+08 ± 1.87E+08	4.76E+08 ± 2.89E+08	3.21E+07 ± 9.46E+06	1.34E+08 ± 4.64E+07	1.25E+08 ± 6.09E+07	5.29	0.019	0.473
[16 rRNA gene copies g^{-1}]	spruce	2.57E+08 ± 2.13E+08	1.29E+08 ± 1.43E+08	1.10E+07 ± 8.63E+06	1.53E+08 ± 6.89E+07	1.28E+08 ± 6.21E+07			
Quantity of *Actinobacteria* [16 rRNA gene copies g^{-1}]	beech	3.91E+08 ± 2.17E+08	2.51E+08 ± 1.22E+08	1.48E+07 ± 6.77E+06	6.52E+07 ± 1.68E+07	8.23E+07 ± 3.85E+07	5.47	0.023	0.522
	spruce	4.09E+08 ± 2.08E+08	2.66E+08 ± 1.07E+08	1.28E+07 ± 6.25E+06	9.65E+07 ± 1.41E+07	7.87E+07 ± 4.32E+07			
Oxalate	beech	5.10 ± 3.76	3.73 ± 2.05	2.37 ± 0.42	1.57 ± 0.54	8.42 ± 19.49	4.52	0.03	0.607
[mg kg^{-1}]	spruce	1.34 ± 0.40	1.93 ± 0.52	2.14 ± 0.59	1.81 ± 0.49	0.90 ± 0.36			
Acetate	beech	4.08 ± 0.91	7.91 ± 7.55	1.98 ± 1.89	1.78 ± 2.08	1.10 ± 0.62	4.38	0.032	0.632
[mg kg^{-1}]	spruce	2.04 ± 1.57	9.92 ± 10.06	3.37 ± 1.98	3.63 ± 6.02	1.12 ± 1.36			
Malate	beech	0.77 ± 0.45	2.85 ± 4.04	1.68 ± 1.06	0.76 ± 0.26	0.57 ± 0.14	4.35	0.033	0.641
[mg kg^{-1}]	spruce	0.36 ± 0.40	0.84 ± 0.99	0.75 ± 0.72	0.46 ± 0.41	0.20 ± 0.15			
NH_4^+	beech	19.5 ± 3.3	54.8 ± 23.0	14.2 ± 5.9	6.79 ± 2.27	3.25 ± 1.76	4.27	0.034	0.652
[mg kg^{-1}]	spruce	13.6 ± 5.7	34.7 ± 18.9	12.2 ± 2.8	4.56 ± 1.09	1.80 ± 0.66			
iAR	beech	19.5 ± 3.3	54.8 ± 23.0	14.2 ± 5.9	6.79 ± 2.27	3.25 ± 1.76	4.12	0.038	0.684
	spruce	13.6 ± 5.7	34.7 ± 18.9	12.2 ± 2.8	4.56 ± 1.09	1.80 ± 0.66			
total LMMOA	beech	28.4 ± 12.8	35.1 ± 25.1	84.2 ± 62.8	24.3 ± 34.5	17.7 ± 21.2	4.01	0.041	0.702
[mg kg^{-1}]	spruce	8.96 ± 5.93	128 ± 117	50.8 ± 31.3	22.4 ± 13.5	11.7 ± 14.6			
arylsulphatase	beech	3.057 ± 2.992	2.312 ± 0.896	2.226 ± 1.616	1.072 ± 0.548	0.655 ± 0.142	3.67	0.051	0.783
[nmol min^{-1} g^{-1}]	spruce	2.150 ± 1.063	1.199 ± 0.533	1.073 ± 0.808	0.256 ± 0.135	0.700 ± 0.618			
SO_4^{2-}	beech	13.5 ± 4.6	53.8 ± 28.7	27.6 ± 6.6	15.0 ± 5.7	21.4 ± 5.2	3.14	0.073	0.884
[mg kg^{-1}]	spruce	21.4 ± 27.4	39.2 ± 14.4	41.5 ± 15.1	38.5 ± 13.5	35.2 ± 24.0			
iDEC	beech	0.73 ± 0.08	0.99 ± 0.16	1.54 ± 0.25	2.07 ± 0.27	2.32 ± 0.51	2.92	0.086	0.917
	spruce	0.76 ± 0.09	0.86 ± 0.15	1.31 ± 0.25	1.61 ± 0.19	1.94 ± 0.40			
Simpson evenness index	beech	0.853 ± 0.023	0.865 ± 0.022	0.833 ± 0.008	0.841 ± 0.011	0.844 ± 0.023	2.7	0.102	0.946
	spruce	0.849 ± 0.033	0.856 ± 0.014	0.839 ± 0.031	0.805 ± 0.029	0.821 ± 0.02			
Formate	beech	1.70 ± 1.78	13.6 ± 22.4	7.86 ± 20.6	8.73 ± 22.21	0.50 ± 0.76	2.65	0.107	0.953
[mg kg^{-1}]	spruce	0.23 ± 0.14	42.5 ± 51.6	17.1 ± 26.6	2.53 ± 3.20	5.90 ± 14.1			
Lactate	beech	10.8 ± 3.9	11.2 ± 7.7	6.85 ± 3.52	6.37 ± 7.92	4.05 ± 1.20	2.56	0.126	0.973
[mg kg^{-1}]	spruce	1.97 ± 3.58	8.82 ± 5.01	5.54 ± 3.47	3.02 ± 0.85	1.63 ± 1.36			
Quantity of fungi	beech	1.42E+08 ± 1.19E+08	5.57E+07 ± 4.24E+07	2.11E+06 ± 1.21E+06	8.50E+06 ± 5.81E+06	3.12E+07 ± 3.51E+07	2.42	0.127	0.976
[ITS1 copies g^{-1}]	spruce	1.15E+08 ± 9.34E+07	1.64E+07 ± 1.30E+07	7.45E+05 ± 6.90E+05	1.21E+07 ± 4.08E+06	9.15E+06 ± 9.90E+06			

Quantity of PKS type II genes [gene copies g^{-1}]	beech	1.90E+06 ± 2.16E+06	5.95E+06 ± 8.29E+06	2.19E+05 ± 1.25E+05	6.60E+05 ± 6.30E+05	3.53E+06 ± 4.64E+06	2.42	0.14	0.987
	spruce	5.14E+06 ± 5.03E+06	3.96E+06 ± 4.01E+06	5.63E+05 ± 1.11E+06	1.35E+06 ± 4.17E+05	2.35E+06 ± 3.69E+06			
Isovalerate [mg kg^{-1}]	beech	0.15 ± 0.07	0.35 ± 0.41	0.16 ± 0.10	0.07 ± 0.06	0.09 ± 0.09	1.84	0.211	0.999
	spruce	0.05 ± 0	0.07 ± 0.06	0.04 ± 0	0.04 ± 0	0.04 ± 0			
SOC [%]	beech	33.2 ± 5.0	27.5 ± 5.5	19.5 ± 4.4	10.2 ± 1.8	5.28 ± 3.17	1.16	0.425	1
	spruce	28.0 ± 5.0	30.3 ± 4.2	21.6 ± 5.1	11.5 ± 2.4	4.05 ± 1.76			
acid phosphatase [nmol min^{-1} g^{-1}]	beech	487.9 ± 129.3	389.0 ± 125.4	117.0 ± 24.4	67.27 ± 25.1	21.36 ± 8.76	1.15	0.407	1
	spruce	299.2 ± 61.6	231.6 ± 60.3	118.2 ± 55.7	45.57 ± 13.52	13.45 ± 8.24			
β-D-glucosidase [nmol min^{-1} g^{-1}]	beech	267.3 ± 87.5	101.4 ± 54.1	12.63 ± 5.82	3.969 ± 1.586	2.389 ± 1.375	1.5	0.29	1
	spruce	237.0 ± 96.7	85.54 ± 31.33	19.46 ± 11.58	5.380 ± 1.399	2.555 ± 2.590			
lipase [nmol min^{-1} g^{-1}]	beech	461.6 ± 58.3	522.5 ± 132.0	175.9 ± 41.6	92.40 ± 25.97	75.48 ± 23.69	1.23	0.378	1
	spruce	384.8 ± 122.1	418.1 ± 61.4	225.8 ± 69.7	95.15 ± 13.7	69.72 ± 29.65			
cellobiohydrolase [nmol min^{-1} g^{-1}]	beech	104.6 ± 69.2	24.94 ± 16.36	1.091 ± 0.998	0.463 ± 0.262	0.445 ± 0.321	1.79	0.221	1
	spruce	105.0 ± 69.8	24.27 ± 16.77	4.696 ± 3.593	0.695 ± 0.494	0.327 ± 0.507			
leucine aminopeptidase [nmol min^{-1} g^{-1}]	beech	1.682 ± 0.859	1.649 ± 0.620	0.659 ± 0.244	0.401 ± 0.159	0.272 ± 0.238	1.49	0.292	1
	spruce	0.930 ± 0.296	2.416 ± 1.543	0.985 ± 0.421	0.656 ± 0.350	0.157 ± 0.098			

S3 Table. Differences between beech and spruce forest soils across all horizons for soil characteristics calculated per square meter. Hotteling's two sample test is used to compare the beech and spruce stands. Permutation of sites is used to adjust the p-values for the multiple comparison problem.

**Soil characteristics**	**T2 stat.**	**P**	P_{adj}
Si	26.48	<0.001	0.003
Quinate	20.26	<0.001	0.007
P	14.37	0.001	0.019
Quantity *erm* genes	14.26	0.001	0.019
NO_3^-	13.95	0.001	0.02
Al	10.52	0.002	0.041
Citrate	7.04	0.008	0.147
Quantity of bacteria	6.51	0.011	0.194
Fe	6.48	0.011	0.198
Ca	5.23	0.02	0.314
PO_4^{3-}	4.8	0.025	0.374
K	3.52	0.056	0.616
S	3.47	0.058	0.625
Acetate	3.35	0.063	0.658
Malate	3.29	0.066	0.676
Quantity of fungi	3.2	0.07	0.7
Formate	2.95	0.084	0.761
DOC	2.93	0.086	0.765
Mg	2.85	0.091	0.785
total LMMOA	2.71	0.101	0.831
SOC	2.65	0.106	0.84
Quantity of *Actinobacteria*	2.24	0.149	0.916
Isovalerate	2.11	0.166	0.938
Oxalate	1.74	0.232	0.982
Lactate	1.58	0.268	0.99
SO_4^{2-}	1.43	0.311	0.997
Quantity of PKS type II genes	1.17	0.4	0.999
NH_4^+	0.82	0.566	1

S4 Table. Statistics supporting the correlation of linear vectors representing chemical variables and quantities within the ordination space of soil prokaryotic communities (A), and vectors of relative abundances of bacterial phyla and quantities within the ordination space of soil chemical properties (B) as presented in Fig. 2. The vectors were fitted to the NMDS plots using envfit function from the vegan package in R software environment.

A. Chemical variables and quantities *vs*. prokaryotic communities

Soil characteristics	Dim1	Dim2	R^2	P	
pH	-0.1296	0.99157	0.5975	0.0001	***
moisture	-0.88732	0.46116	0.5019	0.0001	***
NH_4^+	-0.91149	0.41133	0.1785	0.0017	**
F^-	-0.81364	0.58137	0.031	0.3739	
Cl^-	-0.99116	-0.13268	0.0731	0.0861	.
NO_2^-	-0.92928	-0.36939	0.0301	0.3766	
NO_3^-	-0.41209	-0.91114	0.0323	0.3574	
SO_4^{2-}	0.02773	-0.99962	0.0406	0.2637	
Quinate	-0.31729	-0.94833	0.3222	0.0001	***
Lactate	-0.64603	0.76331	0.117	0.0169	*
Acetate	-0.97305	0.23058	0.1247	0.011	*
Propionate	-0.41063	0.9118	0.0389	0.2813	
Formate	-0.39876	-0.91706	0.0526	0.1775	
Isobutyrate	-0.96243	-0.27154	0.0477	0.2036	
Butyrate	-0.9011	-0.43361	0.0376	0.2879	
Pyruvate	-0.66968	0.74265	0.0088	0.7738	
Isovalerate	-0.25951	0.96574	0.0685	0.0938	.
Adipate	-0.98374	-0.1796	0.1962	0.0007	***
Malate	-0.38007	0.92496	0.0053	0.8645	
Oxalate	-0.03437	0.99941	0.0328	0.3827	
Citrate	-0.93166	-0.36334	0.0035	0.9065	
Al	0.1607	-0.987	0.2472	0.0003	***
Ca	-0.53214	0.84666	0.2112	0.0003	***
Fe	-0.07302	-0.99733	0.3882	0.0001	***
K	-0.96876	0.24798	0.1558	0.0036	**
Mg	-0.3465	0.93805	0.0233	0.4729	
Mn	-0.4621	0.88683	0.2133	0.0002	***
Na	-0.2689	-0.96317	0.0572	0.1462	
Ni	-0.90976	-0.41514	0.0012	0.9609	
PO_4^{3-}	-0.9895	-0.14451	0.2392	0.0002	***
S	-0.303	-0.95299	0.0966	0.0382	*
Si	-0.2609	-0.96537	0.4581	0.0001	***
DOC	-0.44571	-0.89518	0.1062	0.0265	*
quantity of bacteria	-0.723	0.69084	0.5079	0.0001	***
quantity of fungi	-0.63696	0.7709	0.4264	0.0001	***
quantity of actinomycetes	-0.8689	0.49498	0.5077	0.0001	***
quantity of type II PKS genes	-0.6999	0.71424	0.0245	0.4654	
quantity of *erm* genes	-0.29812	-0.95453	0.0865	0.0524	.
PWI	-0.98051	-0.19645	0.8723	0.0001	***
iAR	-0.95606	-0.29317	0.876	0.0001	***
iDEC	0.99444	0.10534	0.7762	0.0001	***
InvSimpson	-0.40222	-0.91554	0.1371	0.0086	**
Evenness	-0.83542	-0.54961	0.1376	0.0083	**
Rf area	0.65682	0.75404	0.3419	0.0001	***
SOC	-0.99994	-0.01118	0.7232	0.0001	***

B. Relative abundances of bacterial phyla and quantities *vs*. soil chemical properties

Community characreristics	Dim1	Dim2	r2	Pr(>r)	
quantity of bacteria	-0.0255	0.99967	0.338	0.0001	***
quantity of fungi	-0.14853	0.98891	0.3224	0.0001	***
quantity of actinomycetes	-0.01637	0.99987	0.3919	0.0001	***
quantity of type II PKS genes	-0.02922	0.99957	0.0188	0.5283	
quantity of *erm* genes	-0.75871	0.65143	0.02	0.5068	
Pseudomonadota	0.72059	0.69336	0.4469	0.0001	***
Actinomycetota	-0.00992	0.99995	0.5024	0.0001	***
Chloroflexota	-0.54816	-0.83638	0.5413	0.0001	***
Acidobacteriota	-0.07428	-0.99724	0.4242	0.0001	***
Ca. Eremiobacterota	0.9685	-0.24901	0.1443	0.0058	**
Thermoproteota	-0.43695	-0.89949	0.5397	0.0001	***
Planctomycetota	0.99396	-0.10973	0.2963	0.0001	***
RCP2-54	0.02619	-0.99966	0.3305	0.0001	***
Bacteroidota	0.03707	0.99931	0.3195	0.0001	***
Bacillota	-0.53816	-0.84284	0.3915	0.0001	***
Verrucomicrobiota	0.45805	-0.88893	0.3119	0.0001	***
Gemmatimonadota	-0.36141	-0.93241	0.5152	0.0001	***
Myxococcota	0.90733	-0.42043	0.0615	0.1208	

S5 Table. Comparison of prokaryotic communities. AMOVA based on Bray-Curtis distance matrix (*** $p \leq 0.001$, ** $p \leq 0.01$, * $p \leq 0.05$).

A. Between beech and spruce forest stands

**Comparison**		**Fs**	**p-value**
Overall		12.8796	***
BA	SA	8.76286	**
BB	SB	3.91286	***
BF	SF	8.6098	***
BH	SH	10.3116	***
BL	SL	10.8776	***

B. Between individual horizons in the soil profile

**Comparison**		**Fs**	**p-value**
Overall		12.8796	***
Beech			
BA	BB	7.02652	**
BA	BF	8.81234	***
BA	BH	1.637	
BA	BL	17.4696	***
BB	BF	14.527	***
BB	BH	12.2061	***
BB	BL	21.4133	***
BF	BH	8.00645	***
BF	BL	8.41536	***
BH	BL	19.6802	***
Spruce			
SA	SB	12.768	***
SA	SF	10.7269	***
SA	SH	2.00818	*
SA	SL	17.1377	**
SB	SF	18.9223	***
SB	SH	13.2929	***
SB	SL	18.1654	***
SF	SH	6.91766	***
SF	SL	8.40442	***
SH	SL	14.0482	***

S6 Table. Differences in relationships between the regressors and the responding variables in the two forest soils B (beech) and S (spruce). Linear mixed model with shared random effect calculated across all soil horizons.

**Regressor**	**Responding variable**	**slope B**	**slope S**	**P**	$\mathbf{P_{adj}}$
Quantity of bacteria	Leucine aminopeptidase	1.42	0.88	0.0017	0.023
	DOC	1.12	0.69	0.0036	0.079
	Lipase	1.22	0.98	0.0076	0.106
	Formate	1.34	0.63	0.01	0.221
	Adipate	1.29	0.97	0.0114	0.252
	Cellobiohydrolase	2.23	0.98	0.0142	0.198
	SOC	2.46	-0.81	0.0302	0.665
	iAR	0.02	-0.01	0.0324	0.712
	iDEC	0.88	0.99	0.0357	0.786
	Horizon thickness	0.76	0.98	0.0394	0.868
Quantity of fungi	Lactate	1.11	0.79	0.0014	0.031
	Formate	1.17	0.71	0.0092	0.202
	DOC	0.97	0.74	0.0115	0.252
	Oxalate	1.1	0.98	0.0296	0.652

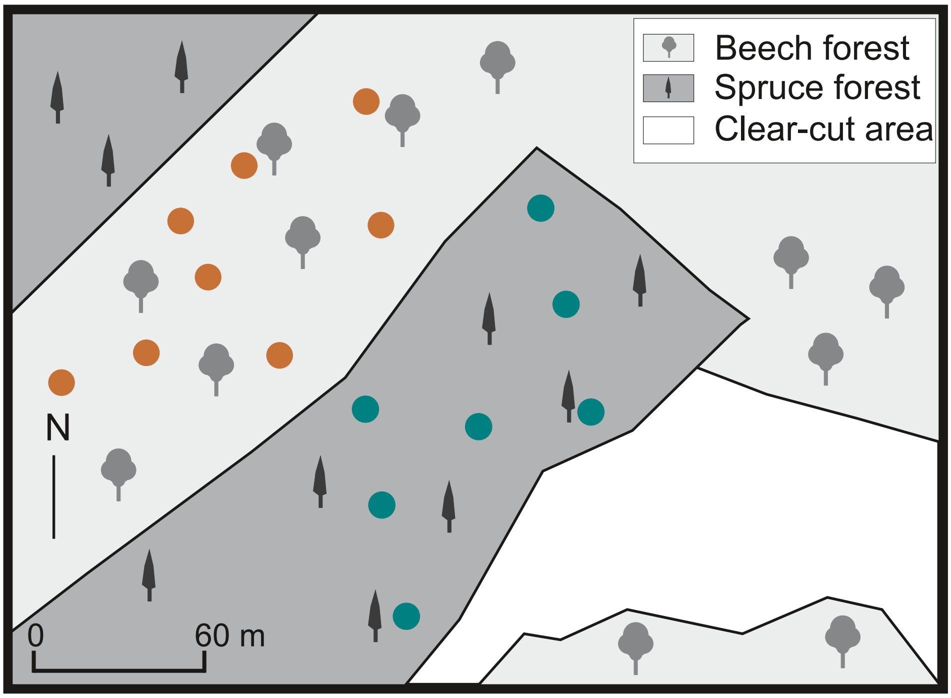

S1 Fig. Sampling sites (map from our own survey).
Beech forest: 50.8677 N 15.2528 E, spruce forest: 50.8673 N 15.2544 E.

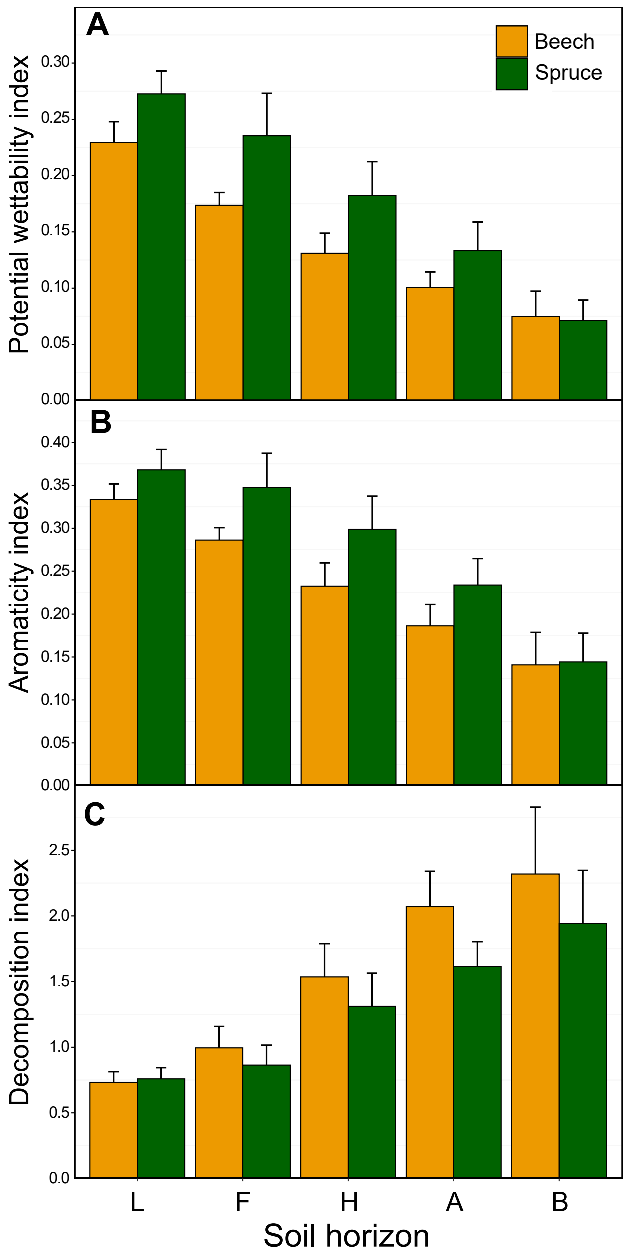

S2 Fig. Potential wettability (A), aromaticity (B) and decomposition (C) indices in soil horizons (L – litter, F – fermentation, H – humified, A – organo-mineral and B – subsurface horizon) under beech (orange) and spruce (dark green) (n = 7).

S3 Fig. Average DRIFT spectra (n = 7) in soil horizons (L - litter, F - fermentation, H - humic, A - organo-mineral and B - subsurface) under beech (blue) and spruce (orange).
Bands of organic soil components are highlighted in green: 1 – C–H stretching in CH_2 a CH_3 groups (2930 a 2850 cm^{-1}), 2 – C=O stretching in carboxylic group (1730–1720 cm^{-1}),
3 – C=O stretching of ketones and amides with aromatic C=C stretching (around 1650 cm^{-1}),
4 – aromatic C=C stretching and N–H stretching of amides (around 1520 cm^{-1}),
5 – C–O stretching of polysaccharides (1125 cm^{-1}) and also C–H of alcohols and carboxyls (1200–1000 cm^{-1}), and 6 – stretching of several groups (aromatic, phenol, carboxyl) including bands around 1370 and 1270 cm^{-1} of lignin.

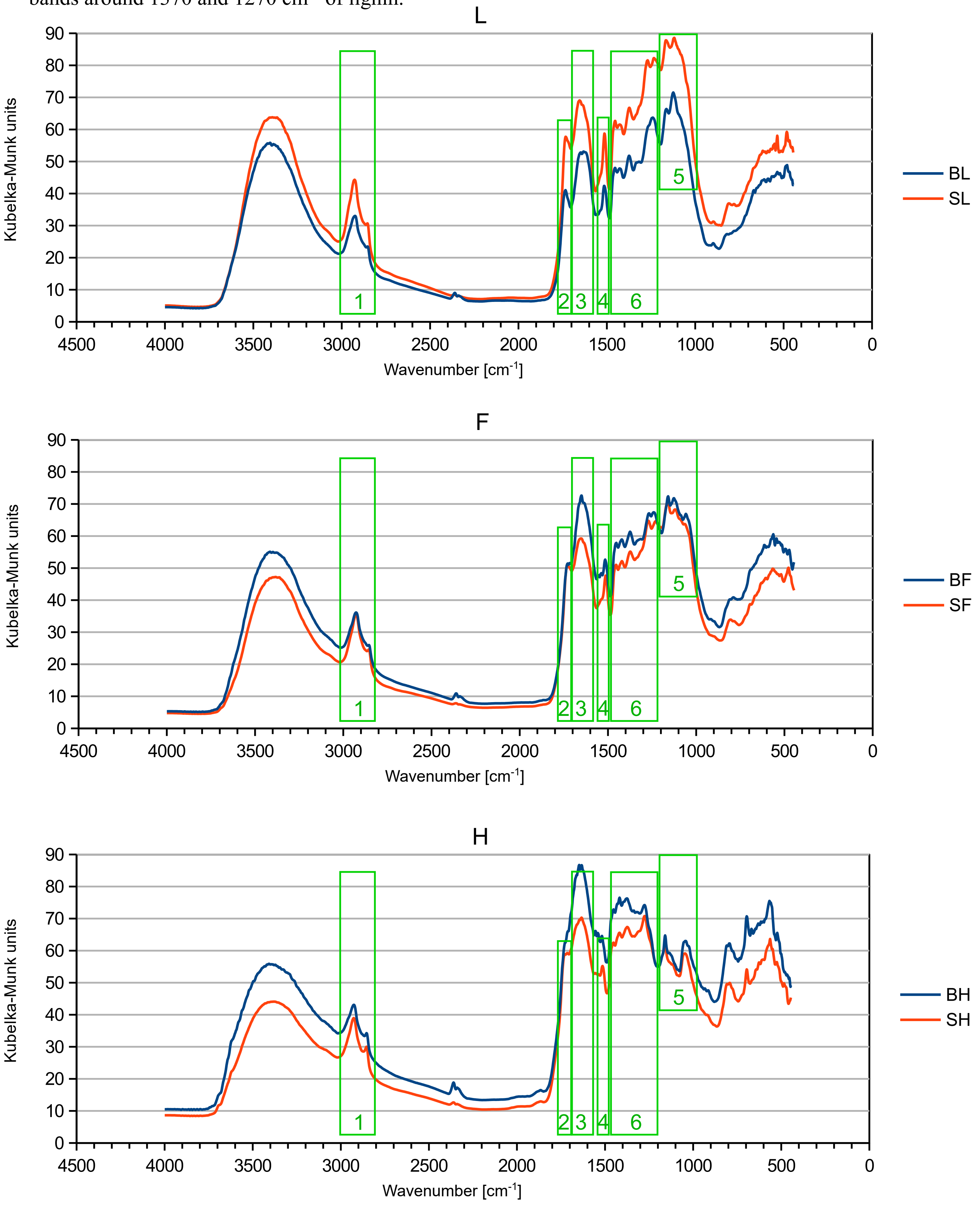

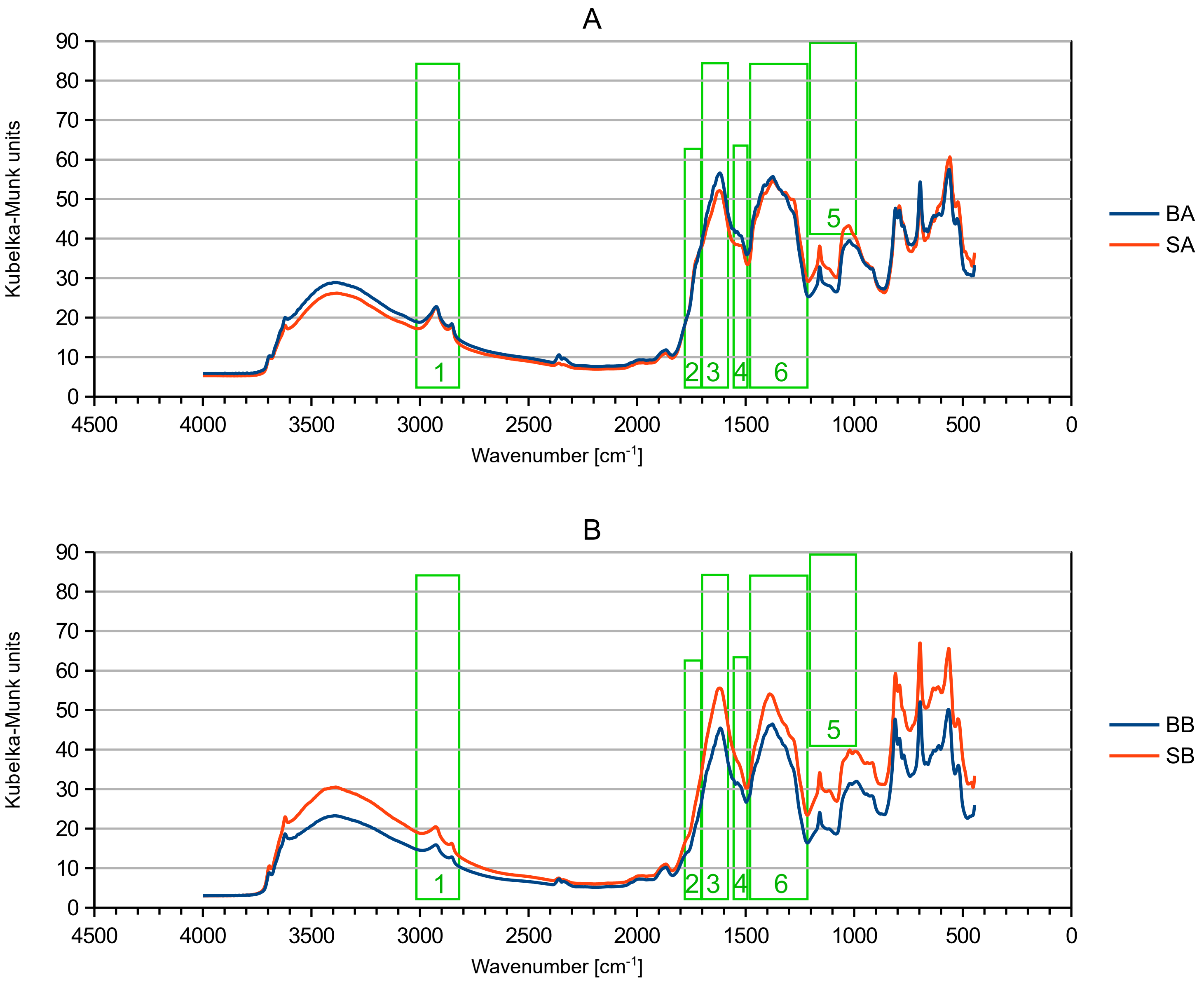
A
B
Kubelka-Munk units
Wavenumber [cm-1]
BA
SA
BB
SB
1
2
3
4
5
6

S4 Fig. Taxonomic assignment (at the phylum level) of the AVSs significantly contributing to the difference between prokaryotic communities in the corresponding horizons of the two forest sites determined by Metastats analysis. Values in the table represent the average percentage in the community (n=7).

A. ASVs significantly increased in the beech forest

	Beech					Spruce				
**Phylum**	**BL**	**BF**	**BH**	**BA**	**BB**	**SL**	**SF**	**SH**	**SA**	**SB**
Pseudomonadota	27.27	30.58	25.1	16.09	10.72	3.04	9.05	2.67	0.8	2.52
Acidobacteriota	4.72	7.08	16.07	17.86	11.64	0.89	0.86	1.73	2.79	2.3
Actinomycetota	15.54	6.96	3.08	3.16	2.16	1.37	1.15	0.69	0.61	0.54
Planctomycetota	1.31	2.09	2.97	1.78	1.84	0.2	0.16	0.27	0.04	0.33
Chloroflexota	1.68	0.15	0.73	1.73	4.56	0	0	0.02	0.25	0.96
Verrucomicrobiota	0.36	1.16	1.69	1.14	0.87	0.04	0.09	0.11	0.04	0.1
Bacteroidota	3.13	0.53	0.48	0.31	0.02	0.28	0.01	0.04	0.02	0
Myxococcota	0.89	0.58	1.15	0.67	0.5	0.02	0.03	0.08	0.01	0.07
RCP2-54	0	0.01	1.03	1.19	0.19	0	0	0.26	0.33	0.04
Ca. Eremiobacterota	0	0.87	1.2	0.84	0	0	0.04	0.04	0.02	0
Gemmatimonadota	0.04	0.02	0.6	0.54	0.68	0	0	0.02	0.02	0.15
uncl. *Bacteria*	0.44	0.01	0.35	0.39	0.13	0	0	0	0.02	0.04
Thermoproteota	0	0.02	0.35	0.64	0.19	0	0	0	0.03	0.03
Armatimonadota	0.4	0.09	0.03	0.02	0.02	0.04	0.01	0	0	0
WPS-2	0.33	0	0	0	0.05	0.01	0	0	0	0.01
SAR324_clade	0	0.08	0.11	0.08	0.02	0	0	0	0	0
Dependentiae	0.01	0.07	0.1	0.04	0.02	0	0	0.01	0	0.01
Elusimicrobiota	0	0	0.08	0.05	0.12	0	0	0	0	0.01
Bacillota	0	0	0.03	0	0.15	0	0	0	0	0.01
Cyanobacteriota	0.04	0.06	0.06	0.03	0	0	0	0	0	0
Thermoplasmatota	0	0	0.04	0.03	0.07	0	0	0	0	0
Nitrospirota	0	0	0	0	0.1	0	0	0	0	0.01
Patescibacteria	0.03	0	0.02	0.03	0.01	0	0	0	0	0
FCPU426	0	0	0.05	0.02	0	0	0	0	0	0
GAL15	0	0	0	0	0.04	0	0	0	0	0.02
NB1-j	0	0	0	0	0.06	0	0	0	0	0
Bdellovibrionota	0	0	0.03	0	0	0	0	0	0	0
Fibrobacterota	0	0.01	0	0	0	0	0	0	0	0
MBNT15	0	0	0	0	0.01	0	0	0	0	0

B. ASVs significantly increased in the spruce forest

	Beech					Spruce				
**Phylum**	**BL**	**BF**	**BH**	**BA**	**BB**	**SL**	**SF**	**SH**	**SA**	**SB**
Actinomycetota	5.16	5.2	2.65	4.52	0.91	36.49	23.21	8.02	12.79	3.01
Pseudomonadota	2.69	2.39	2.69	3.68	1.61	11.95	16.05	21.2	26.44	5.18
Acidobacteriota	0.66	1	0.92	0.92	1.09	7.76	7.98	12.83	10.31	5.63
Chloroflexota	0.02	0.33	0.12	0.18	3.92	0.67	2.54	0.49	1.17	11.41
Ca. Eremiobacterota	0.28	0.1	0.61	0.27	0	2.3	2.5	4.16	2.43	0
Planctomycetota	0.12	0.29	0.51	0.11	0.16	1.97	2.98	2.37	1.46	0.97
RCP2-54	0	0	0.58	0.66	0.14	0.04	0.18	3.8	3.17	0.49
Verrucomicrobiota	0.02	0.11	0.14	0.16	0.07	0.38	1.07	1.14	1.37	0.38
Bacteroidota	0.04	0.03	0.02	0	0	0.44	0.88	0.49	0.36	0.04
Crenarchaeota	0.02	0.05	0	0.04	0.26	0.36	0.34	0.06	0.2	0.7
Gemmatimonadota	0	0.01	0.03	0	0.06	0.07	0.3	0.11	0.09	0.54
WPS-2	0	0	0	0	0.02	0	0	0	0	0.86
uncl. *Bacteria*	0	0	0	0	0.16	0.01	0	0	0	0.69
Myxococcota	0.01	0	0.02	0.01	0.01	0.03	0.16	0.15	0.29	0.12
Bacillota	0	0	0	0	0.06	0.05	0.08	0	0	0.39
Cyanobacteriota	0	0	0	0	0	0.21	0.1	0.05	0.04	0.05
Armatimonadota	0	0	0	0.01	0.02	0.12	0.04	0.02	0.09	0.1
Dependentiae	0	0	0	0	0	0	0.03	0.03	0.12	0.08
GAL15	0	0	0	0	0.02	0	0	0	0	0.1
Patescibacteria	0	0	0	0	0	0.01	0.04	0.01	0.01	0.05
Thermoplasmatota	0	0	0	0	0.01	0	0	0	0.01	0.08
FCPU426	0	0	0	0	0.01	0	0	0	0.06	0.03
Nitrospirota	0	0	0	0	0	0	0	0	0	0.02
Bdellovibrionota	0	0	0	0	0	0	0	0.01	0	0
Elusimicrobiota	0	0	0	0	0	0	0	0	0.01	0

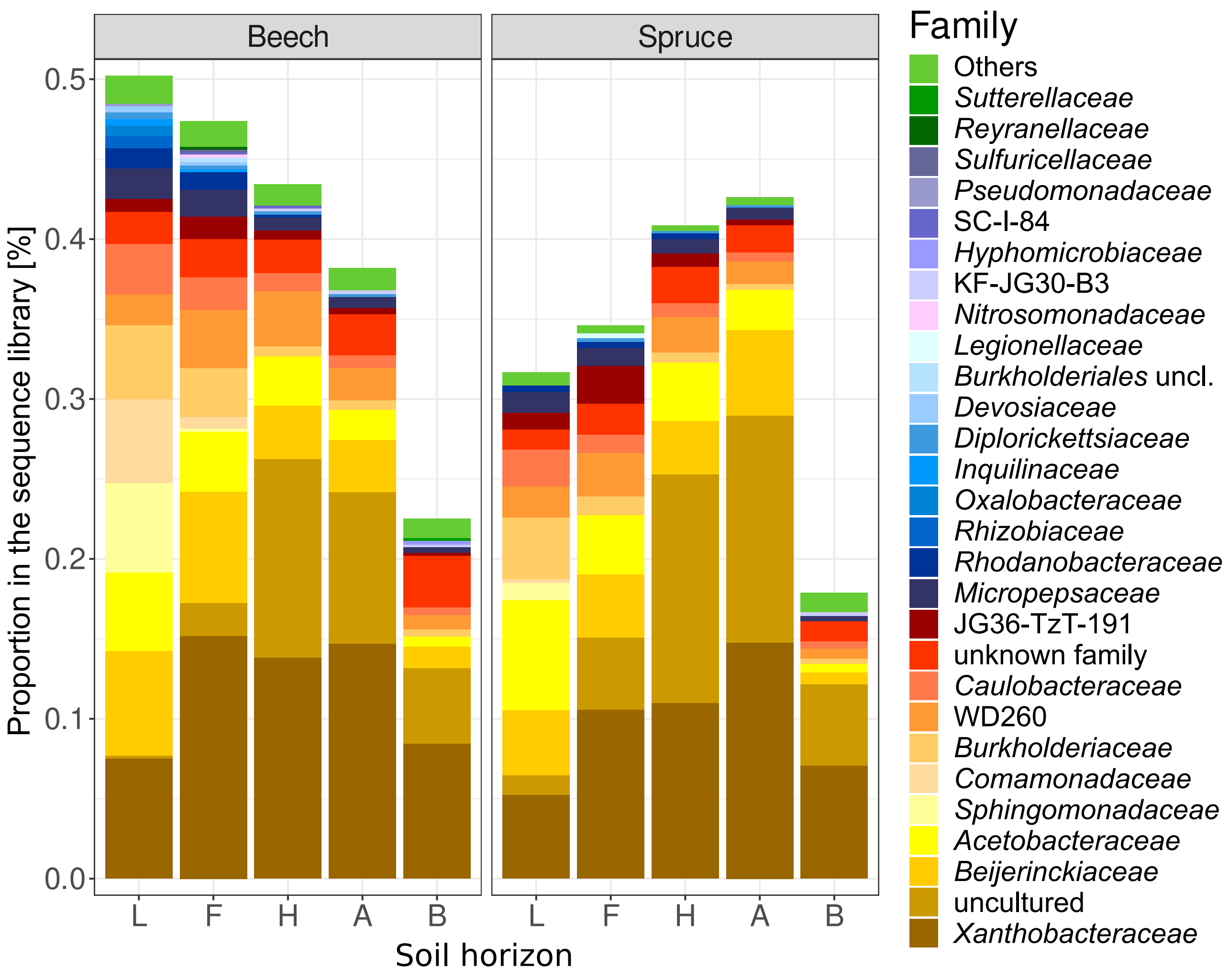

S5 Fig. Proportions the families within the phylum *Pseudomonadota*.

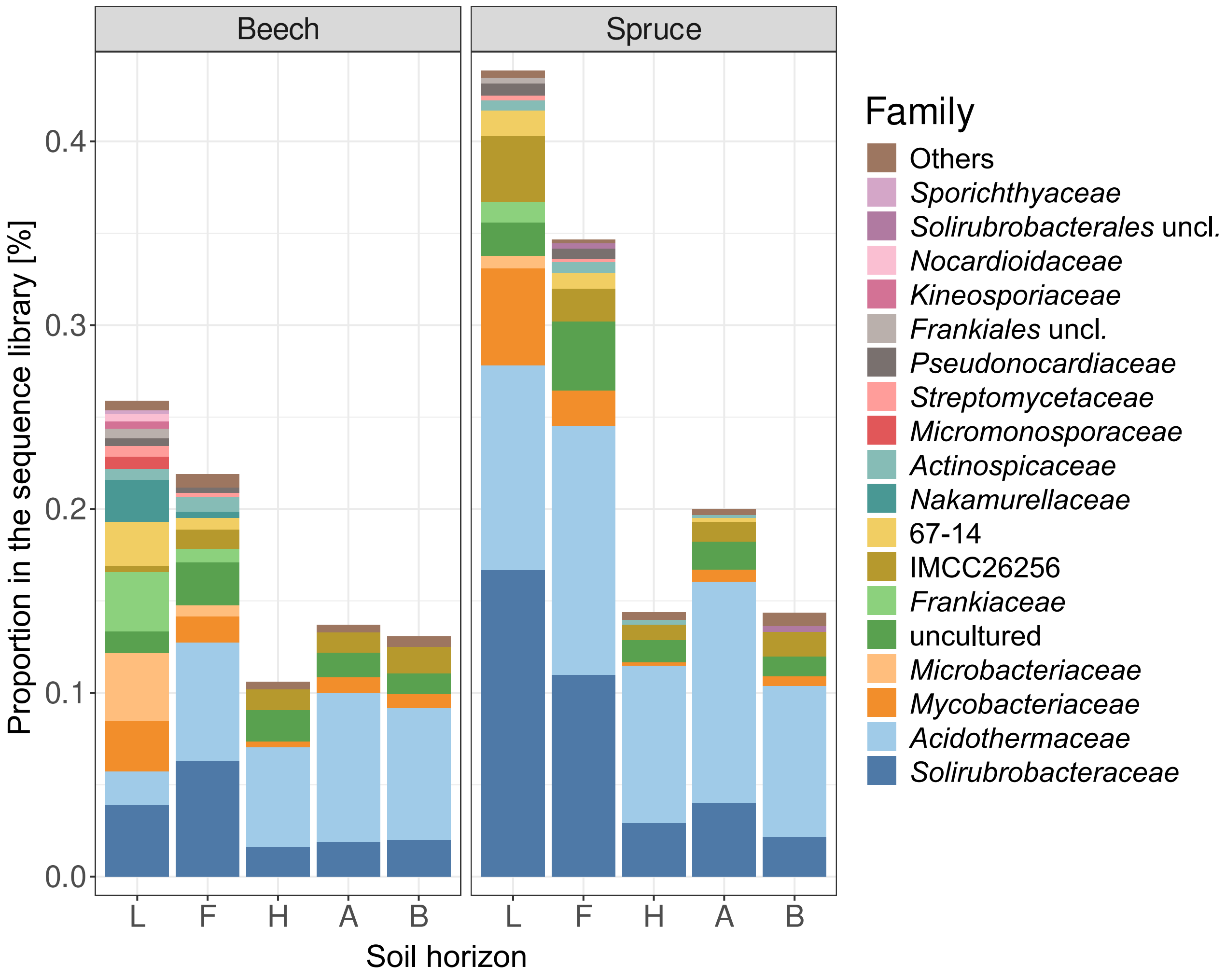

S6 Fig. Proportions the families within the phylum *Actinomycetota*.

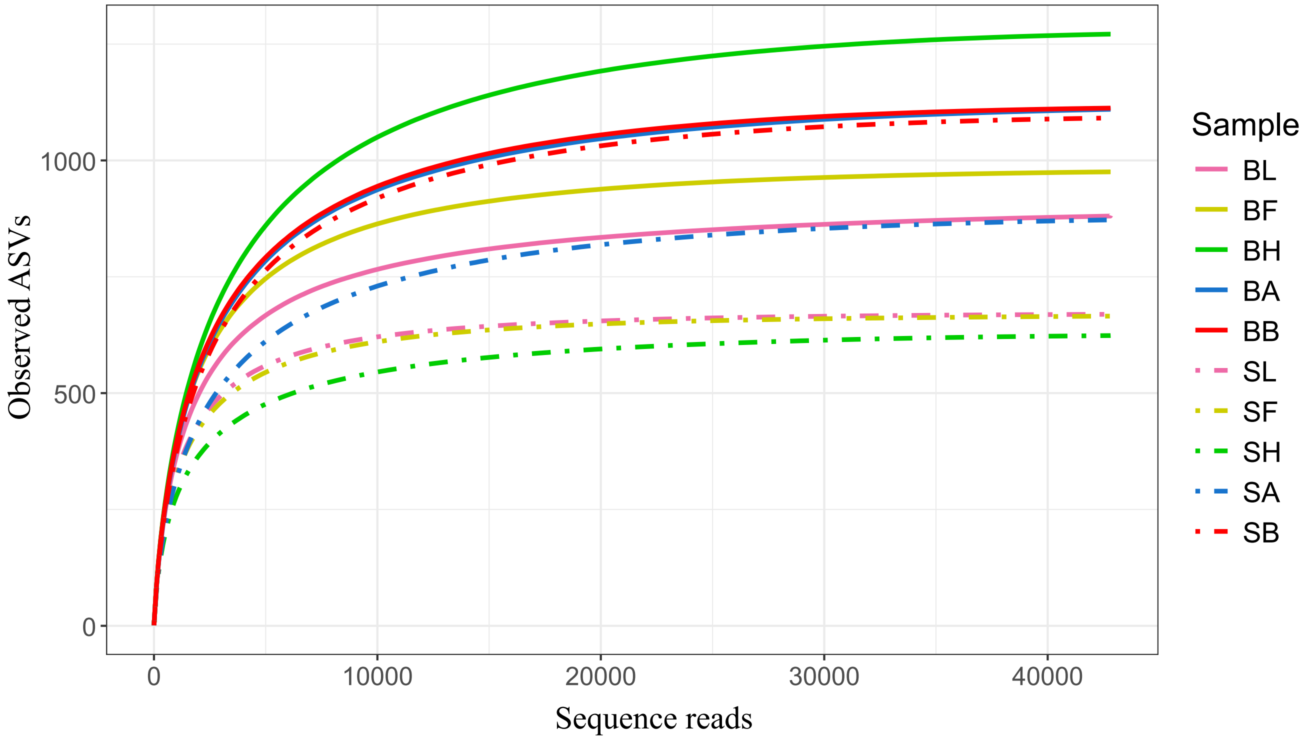

S7 Fig. Estimation of microbial diversity by rarefaction curves in the spruce (S) and beech (B) soil horizons (L -litter, F - fermentation, H - humic, A - organo-mineral and B - subsurface) (n=7).

S8 Fig. Taxonomic assignment of the AVSs significantly contributing to the difference between prokaryotic communities in the corresponding horizons of the two forest sites by their increased proportion in B (left) or S (right) site determined by Metastats analysis ($p < 0.05$).

horizon L

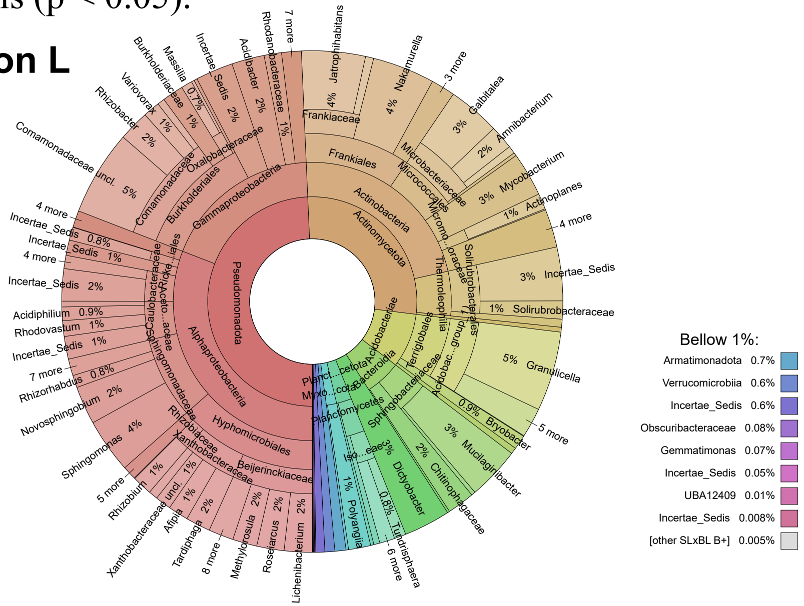

56.18 % of the community
467 ASVs

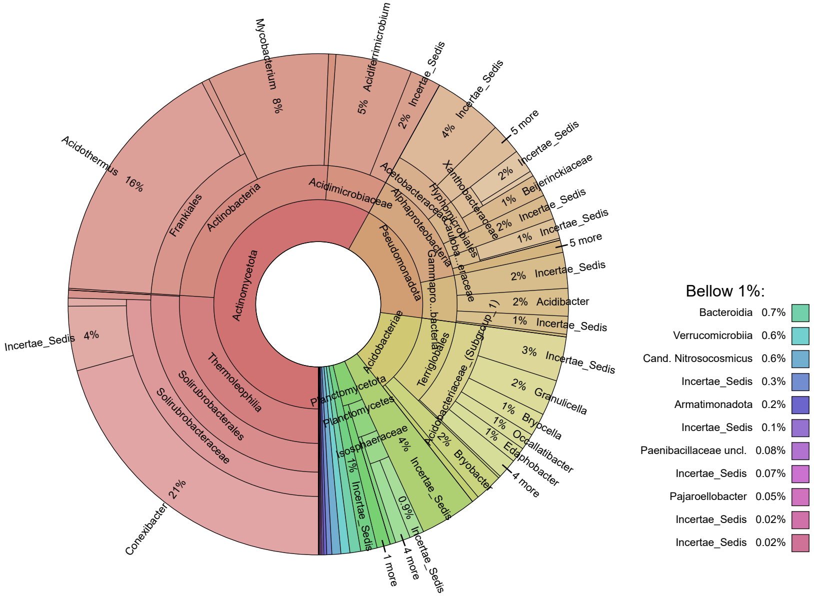

62.85 % of the community
351 ASVs

horizon F

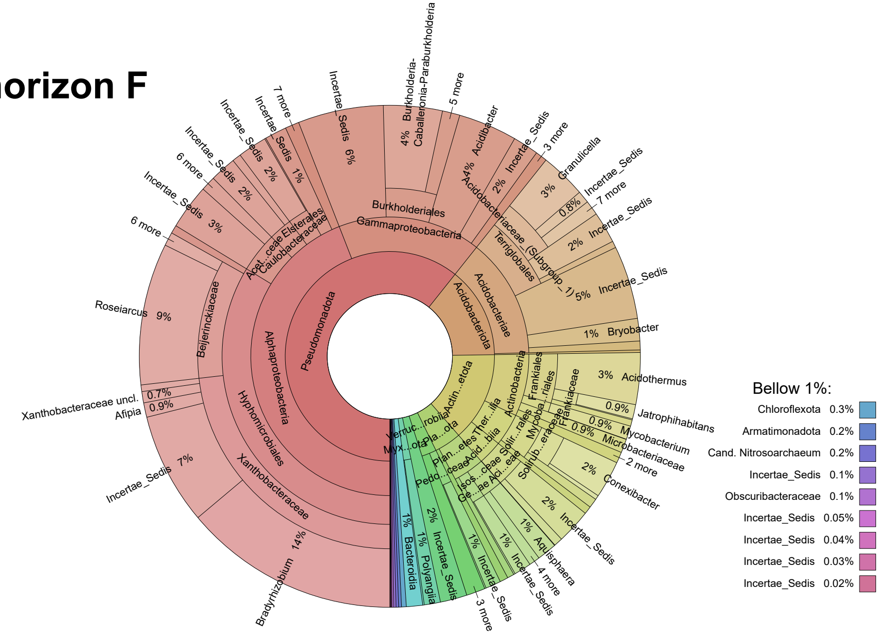

50.39 % of the community
509 ASVs

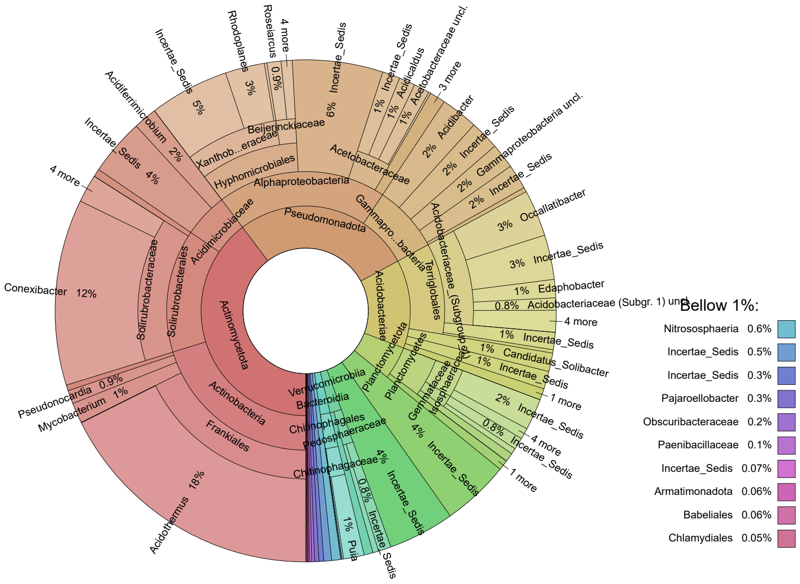

58.49 % of the community
337 ASVs

horizon H

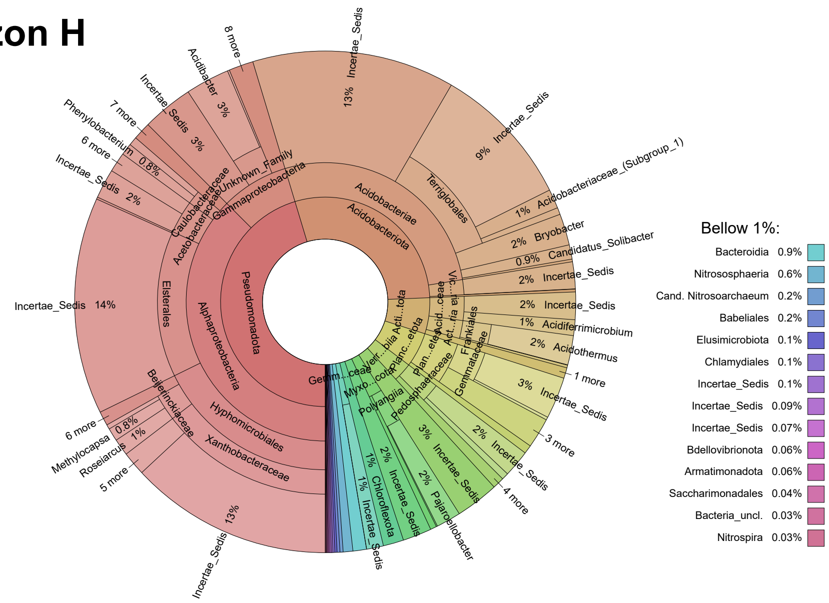

55.36 % of the community
717 ASVs

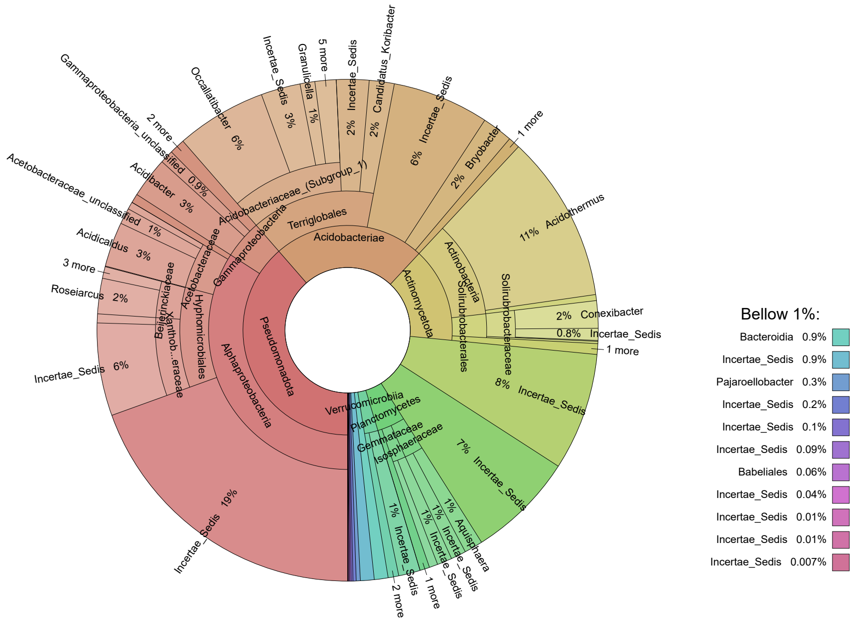

54.95 % of the community
228 ASVs

horizon A

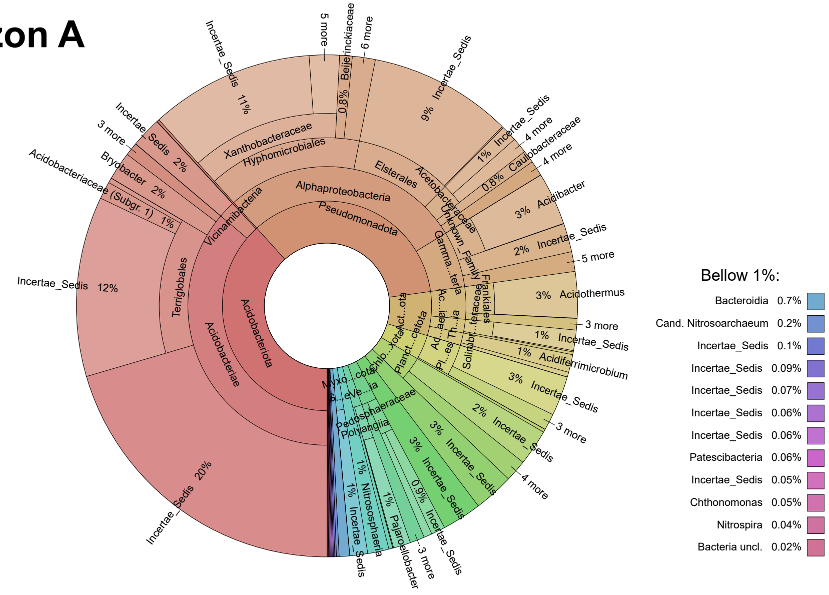

46.64 % of the community
506 ASVs

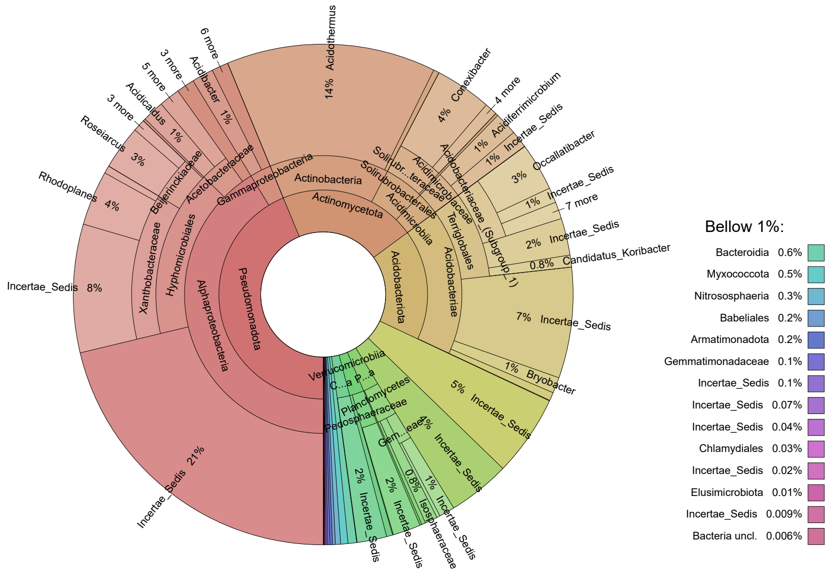

60.42 % of the community
422 ASVs

horizon B

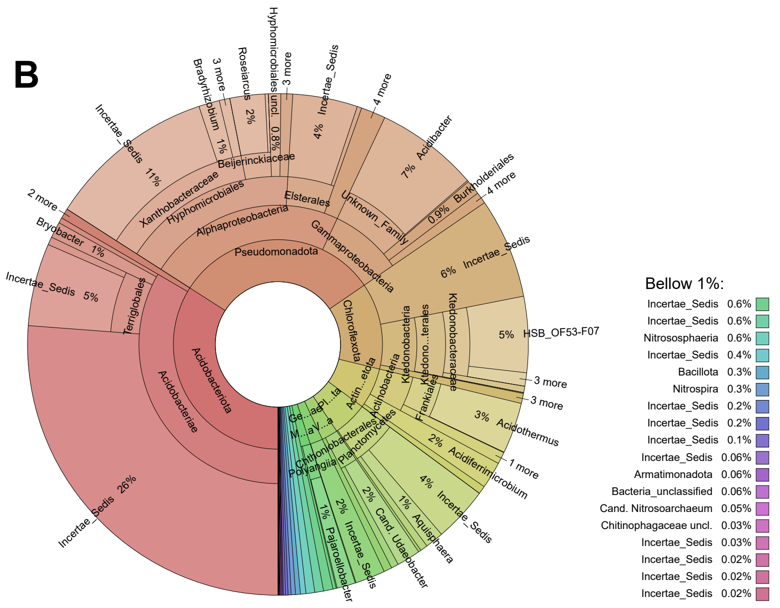

34.16 % of the community
325 ASVs

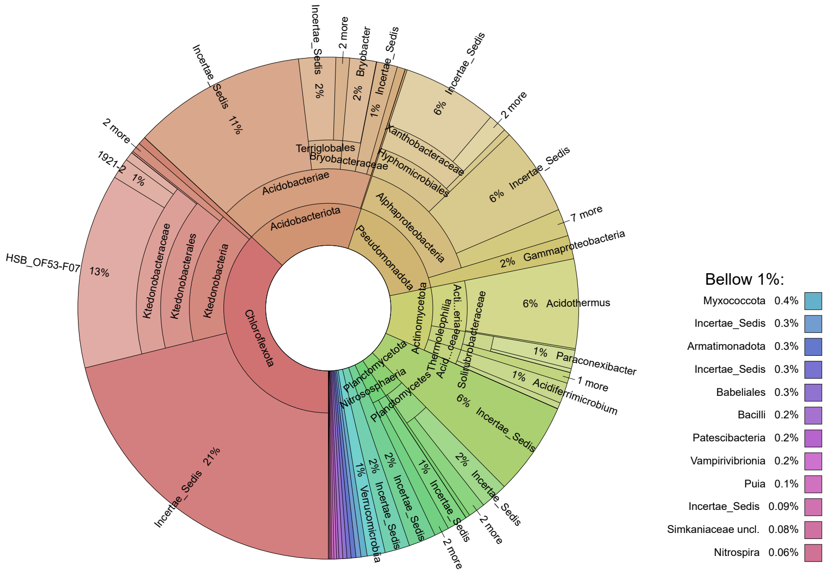

30.92 % of the community
285 ASVs

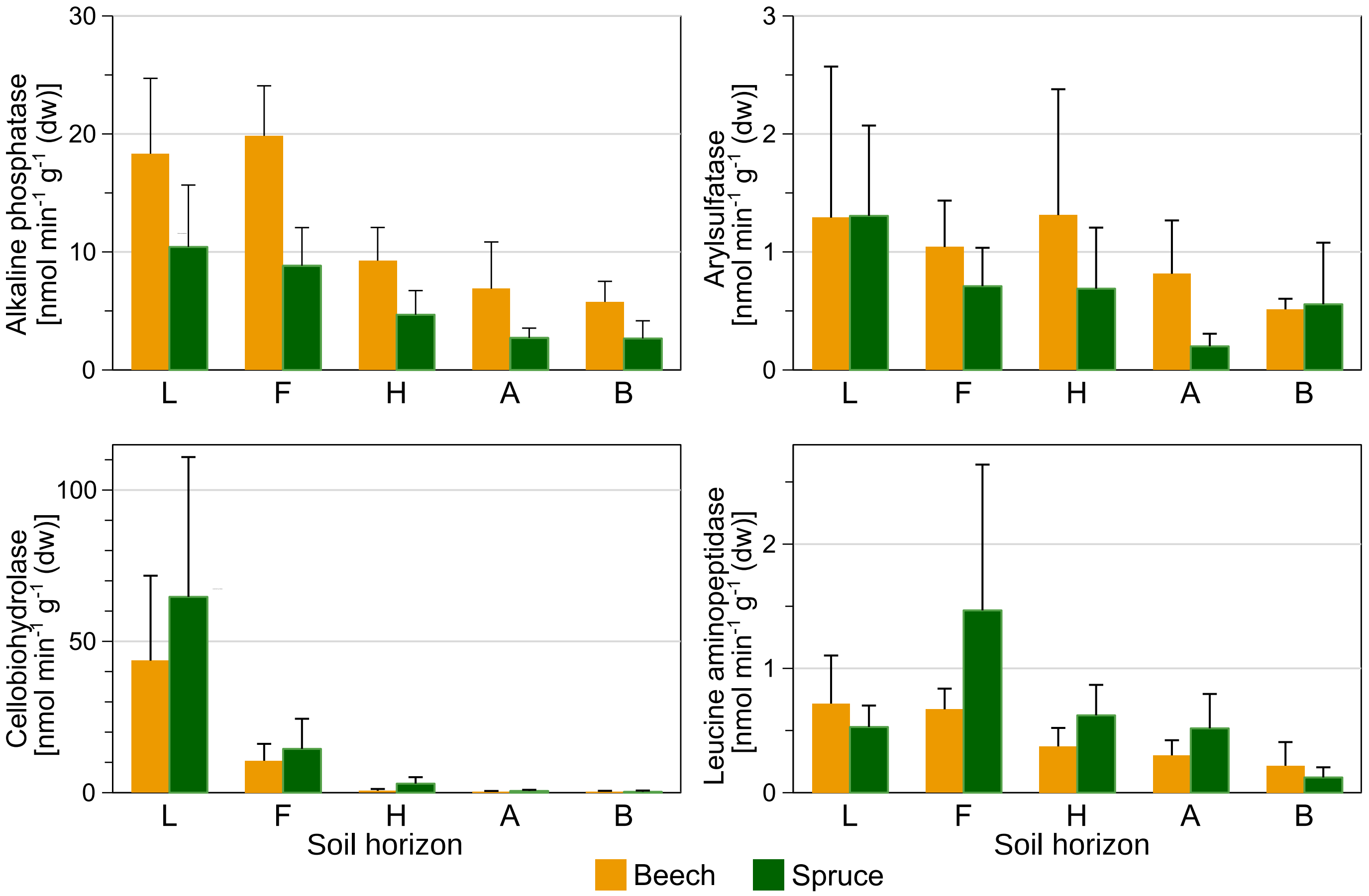

S9 Fig. Activities of extracellular hydrolytic enzymes in soil horizons (L – litter, F – fermentation, H – humified, A – organo-mineral and B – subsurface) under beech (orange) and spruce (dark green) (n = 7).

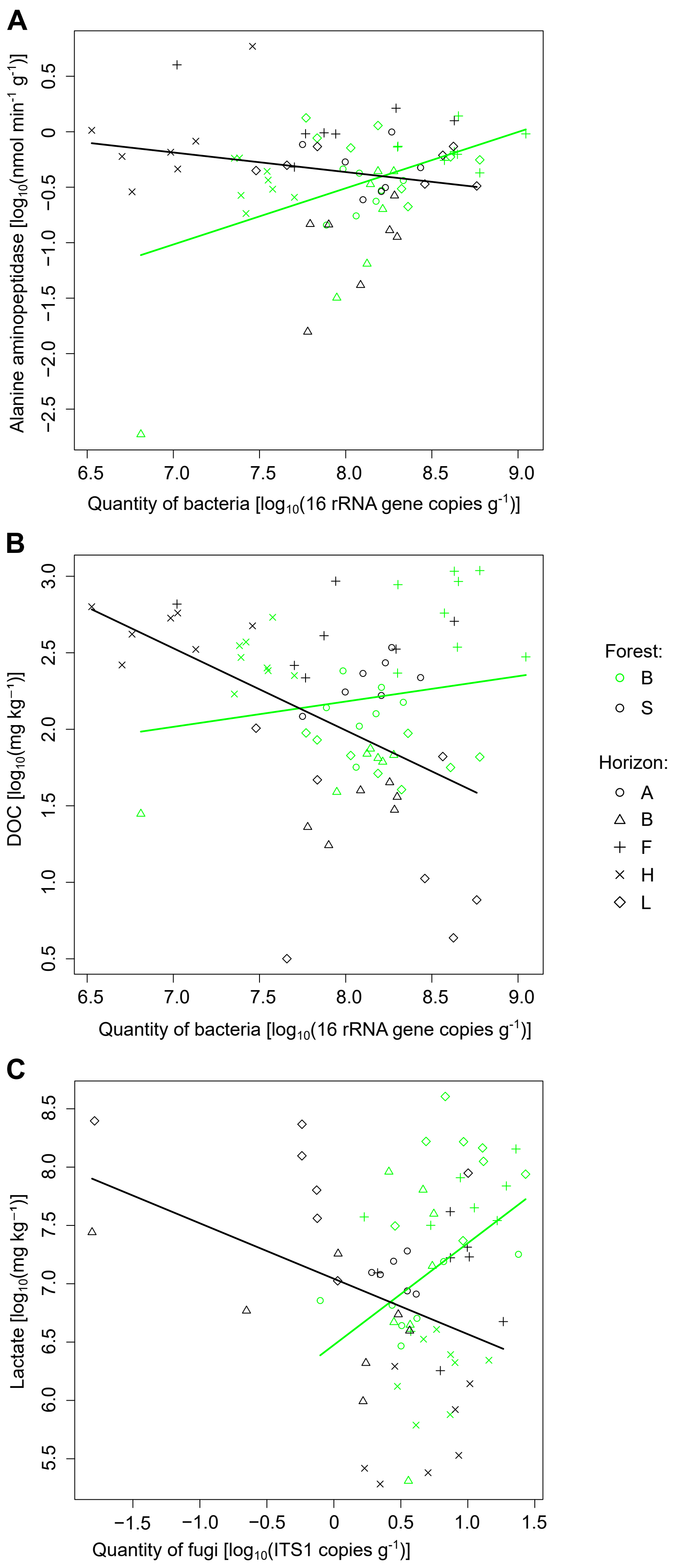

S10 Fig. Relationships between bacterial abundance and leucine aminopeptidase activity (A), and soil DOC content (B), and between fungal abundance and soil lactate content (C). Mixed linear model calculated for beech (B, green) and spruce forest samples (S, black) across all soil horizons (L -litter, F - fermentation, H - humic, A - organo-mineral and B - subsurface) (n=7).

Supplementary references

Buée, M., Reich, M., Murat, C., Morin, E., Nilsson, R.H., Uroz, S., Martin, F., 2009. 454 Pyrosequencing analyses of forest soils reveal an unexpectedly high fungal diversity. New Phytologist 184, 449–456. doi:10.1111/j.1469-8137.2009.03003.x

Čermák, L., Kopecký, J., Novotná, J., Omelka, M., Parkhomenko, N., Plháčková, K., Ságová-Marečková, M., 2008. Bacterial communities of two contrasting soils reacted differently to lincomycin treatment. Applied Soil Ecology 40, 348–358. doi:10.1016/j.apsoil.2008.06.001

Pfeiffer, S., Pastar, M., Mitter, B., Lippert, K., Hackl, E., Lojan, P., Oswald, A., Sessitsch, A., 2014. Improved group-specific primers based on the full SILVA 16S rRNA gene reference database. Environmental Microbiology 16, 2389–2407. doi:10.1111/1462-2920.12350

Sakai, M., Matsuka, A., Komura, T., Kanazawa, S., 2004. Application of a new PCR primer for terminal restriction fragment length polymorphism analysis of the bacterial communities in plant roots. Journal of Microbiological Methods 59, 81–89. doi:10.1016/j.mimet.2004.06.005

Wawrik, B., Kerkhof, L., Zylstra, G.J., Kukor, J.J., 2005. Identification of unique type II polyketide synthase genes in soil. Applied and Environmental Microbiology 71, 2232–2238. doi:10.1128/AEM.71.5.2232-2238.2005

White, J.R., Nagarajan, N., Pop, M., 2009. Statistical methods for detecting differentially abundant features in clinical metagenomic samples. PLoS Computational Biology 5, e1000352. doi:10.1371/journal.pcbi.1000352
