# Supplementary material for: Tree species influence soil carbon quality but not total storage across horizons: European beech on Dystric Cambisol and Norway spruce on Entic Podzol
Source: PLoS One. 2026 Jun 8;21(6):e0350656. doi: 10.1371/journal.pone.0350656 (PMC13245778; doi:10.1371/journal.pone.0350656)
Supplement: Attachment — Submitted filename: review_of_PONE-D-26-03344.pdf [file pone.0350656.s003.pdf]

Manuscript PONE-D-26-03344

Tree species influence carbon quality but not total storage across soil horizons: a comparison of European beech and Norway spruce

Major comments

Thank you for interesting manuscript!

The paper focuses on assessing the influence of two different forest stands (Norway spruce vs. European beech) growing on the same site on forest soil chemistry, element stocks, and microbial communities.

The authors conclude that carbon sequestration and the size of microbial communities do not differ between soils under Norway spruce and European beech stands within the study site. However, they found that most of the studied characteristics are soil- and horizon-specific, which is described in detail in the Results and Discussion sections. The most significant driver appears to be soil pH, which influences decomposition processes and SOC/DOC quality. Another important factor is litter quality, which, in combination with pH differences, influences microbial communities under beech and spruce stands.

I believe that mycorrhizae could also play a crucial role in the acquisition of water and nutrients from the soil, particularly under conditions of low nutrient availability or in acidic soil environments. This represents another important factor that could play a significant role. Especially for tree nutrition.

Another important factor could be the chemistry of the mineral soil, particularly within the rooting zone. This is also documented in the manuscript. However, I did not find any information about the depth of mineral soil (B horizon) sampling.

Overall, the text is well written and clearly explains the background and methodology. The results are well presented and easy to understand. The authors formulated three research questions in the Introduction; therefore, readers might expect explicit answers to these questions at the end of the Discussion or within the Conclusion section, summarizing whether and how the study addressed them.

Minor comments

I have the following minor comments/questions on the paper:

- References are numbered throughout the text; however, in line 54, “Augusto et al. 2002” is cited in text format. Please delete this citation style or renumber the reference list accordingly.
- Pages 99–114: I suggest moving this section to the Materials and Methods. This part describes the methodological procedure. The Introduction could instead conclude with the research questions.
- Section 2.2 Soil sampling: There is no information about the depth of B horizon sampling. How deep were the B horizon samples taken? Alternatively, how thick was the subsoil layer from which the samples were collected?
  Additionally, regarding rock fragment content – you mention that “all sites were about three meters from the closest tree and on a homogeneous substrate (without larger stones).” Does

this mean that larger stones were present at the site, possibly not only on the surface but also within the soil profile? What was the proportion of rock fragments?

- I did not check the completeness of the reference list nor cross-check the references cited in the text. Please review this carefully to avoid inconsistencies.
