# Supplementary material for: Tree species influence soil carbon quality but not total storage across horizons: European beech on Dystric Cambisol and Norway spruce on Entic Podzol
Source: PLoS One. 2026 Jun 8;21(6):e0350656. doi: 10.1371/journal.pone.0350656 (PMC13245778; doi:10.1371/journal.pone.0350656)
Supplement: Attachment — Submitted filename: responsereviewers.docx [file pone.0350656.s005.docx]

Editor

1) The comparison is between two different soil types (Cambisol and Podzol), therefore the effects of tree species are partly confounded with soil type. In several parts, the manuscript attributes differences mainly to tree species without sufficiently discussing pedogenic effects.

*A:Thank you for the comment. The relationship between both tree species and soil type was now elaborated in several parts of the MS including the title.*

2) Hypotheses are vague and not formally tested. Your three research questions are broad and exploratory.

*A:*The hypotheses were changed so they would correspond to the tested relationships.

L114-124

3) The research was conducted at a single locality, which limits generalization of the conclusions. Some statements are formulated too broadly.

*A:*The statements and explanations were modified throughout the MS.

4) Some interpretations, especially regarding secondary metabolites and microbial competition, are rather speculative and should be presented more cautiously.

*A:*This part of MS in Discussion was shortened and speculations removed. Some information on why antibiotics and secondary metabolites are important to study together with decomposition processes was added to the introduction.

L 85-96

5) The Discussion is relatively long and descriptive, and would benefit from a clearer structure and a stronger synthesis of the main findings.

*A:*The discussion was substantially shortened from 1904 words to 1148 words.

6) The manuscript relies on multiple Wilcoxon–Mann–Whitney tests applied separately for each horizon and variable which may raise some concerns.

*A:*The Wilcoxon–Mann–Whitney tests were replaced by Hotteling's two sample test and corrections for multiple testing was done by permutation. This method was recommended by our colleague Marek Omelka Ph.D., who teaches statistics at Charles University and does consulting for environmental and ecological studies.

6) Using Wilcoxon–Mann–Whitney tests applied separately for each horizon and variable may raise concerns about:

- Multiple testing inflation (Type I error)
- Fragmented interpretation
- Lack of integrated multivariate analysis

*A:*Yes, we understand this problem. In the original paper we omitted the Type I error corrections because we did not know how to handle the strong differences between the individual horizons, which in fact were not the focus of the paper as we know that horizons in soil profiles differ. Thus, it seemed that testing for those differences is meaningless. However, our upon your request and with help of our statistician the testing was modified.

See methods.

L 287-291

The authors should:

- Indicate whether any correction for multiple comparisons (e.g. FDR) was applied.
- Consider multivariate approaches (e.g. PERMANOVA for chemistry, dbRDA linking SOC indices to microbial composition).

*A:*Yes. As mentioned above the multiple testing was assessed by permanova now.

- Clarify the structure of the linear mixed models: What exactly was treated as random effect? Were horizons treated as nested within plots? Were assumptions of normality and homoscedasticity checked? The mixed-model results are currently presented briefly and need clearer explanation of model structure, fixed effects, and interaction terms.

Yes, the horizons were treated as nested within plots. The logarithmic transformation was applied were it was useful to bring the data closer to normality and heteroscedasticity assumptions. These assumptions were also checked and seemed to be satisfied in a reasonable way.

*A:*Linear mixed-effects models were used with the measurements from the same
site e.g. B1L, B1F, B1H B1A, B1B, ,sharing the same random effect. These random effects enable the model to account for the variability that comes from the possible dependence of the measurements from the same location.

Reference: Venables, WN, and Ripley BD. Modern Applied Statistics with
S. Springer, New York, NY, 2002.

In addition to Methods:
Seven sampling sites per forest type are mentioned. It should be clearly stated:

- Whether horizons within one soil pit were treated as independent samples in statistical tests.
- How spatial independence between pits was ensured.

Spatial independence was retained by selecting sites with each forest, which were not connected by surface or subsurface water flow, they were about
- Whether forest type is replicated at the landscape level (it appears not).

*A: The sampling was re-written to address these issues. They were further addressed in statistical models.*

The manuscript should clarify the true level of replication to avoid pseudoreplication.

*A: While soil pits can be considered as independent replicates the horizons within one soil pit cannot be treated as independent samples. But this is very naturally incorporated in the used the Hotelling's two sample test which takes the measurements at different horizons within one soil split as a multivariate (5-dimensional) response.*


[Note: HTML markup is below. Please do not edit.]

Reviewer:

Major comments

I believe that mycorrhizae could also play a crucial role in the acquisition of water and nutrients from the soil, particularly under conditions of low nutrient availability or in acidic soil environments. This represents another important factor that could play a significant role. Especially for tree nutrition.

*A: Yes, we agree. However, we did not study mycorrhiza in this work, so we cannot give more specific comments on this issue. Some connections to our results are done by citing literature e.g. the study by Uroz et al. 2016. E.g. L 436,*

Another important factor could be the chemistry of the mineral soil, particularly within the rooting zone. This is also documented in the manuscript. However, I did not find any information about the depth of mineral soil (B horizon) sampling.

*A: The sampling went 25 cm deep in B horizon under both trees. It was not possible to dig deeper because larger stones started to appear. The situation was similar under both trees, so there was not any difference in B horizon thickness.*

*The information is now on*

*L 163.*

Overall, the text is well written and clearly explains the background and methodology. The results are well presented and easy to understand. The authors formulated three research questions in the Introduction; therefore, readers might expect explicit answers to these questions at the end of the Discussion or within the Conclusion section, summarizing whether and how the study addressed them.

*A:* Yes, the answers are now more clearly replied in both Discussion and Conclusions.

Minor comments

I have the following minor comments/questions on the paper:

References are numbered throughout the text; however, in line 54, “Augusto et al. 2002” is cited in text format. Please delete this citation style or renumber the reference list accordingly.

Done.

Pages 99–114: I suggest moving this section to the Materials and Methods. This part describes the methodological procedure. The Introduction could instead conclude with the research questions.

*A: Yes, thank you. We shortened this part only to give a brief methodological introduction to the reader and finished the Introduction with hypotheses. Longer methodological description is provided in Methods.*

*L 105-124*

Section 2.2 Soil sampling: There is no information about the depth of B horizon sampling. How deep were the B horizon samples taken? Alternatively, how thick was the subsoil layer from which the samples were collected? Additionally, regarding rock fragment content – you mention that “all sites were about three meters from the closest tree and on a homogeneous substrate (without larger stones).” Does this mean that larger stones were present at the site, possibly not only on the surface but also within the soil profile? What was the proportion of rock fragments?
*A: The depth of B horizon was about 25 cm, where more skeletal structure occurred. However, on the surface and in the upper horizons there were no stones in the soil. The stones mentioned are rather small rocks, which are randomly dispseudotributed at the site. This was explained in the methods more carefully.*

*L 146-148*

- I did not check the completeness of the reference list nor cross-check the references cited in the text. Please review this carefully to avoid inconsistencies.

*Yes, that was done for this new shorter version.*
