# Supplementary material for: Tree species influence soil carbon quality but not total storage across horizons: European beech on Dystric Cambisol and Norway spruce on Entic Podzol
Source: PLoS One. 2026 Jun 8;21(6):e0350656. doi: 10.1371/journal.pone.0350656 (PMC13245778; doi:10.1371/journal.pone.0350656)
Supplement: Attachment — Submitted filename: Response to Reviewers_0511.docx [file pone.0350656.s006.docx]

**Additional Editor Comments:**

Statistical methods:

Please describe more precisely how the Hotelling’s two-sample test was applied, how the permutation correction for multiple testing was done, and which tests were corrected. Also, please make the terminology consistent. In the response letter you mention PERMANOVA, but in the manuscript the methods refer mainly to Hotelling’s test and AMOVA.

Linear mixed models:
Please clearly describe the model structure: what was the response variable, what were the fixed effects, what was the random effect, and how horizons and sampling sites were treated. Please also briefly state how model assumptions were checked.

Yes, see below an explanation by our statistician Marek Omelka.

Statistical differences of soil variables and enzymatic activities were assessed by Hotelling‘s two-sample test [47]. The individual site were considered independent replicates, while the horizons within one site were treated as a multivariate (5-dimensional) response. The test then compares the multivariate means (each component of the mean corresponding to one horizon) between two samples representing spruce and beech forests. The test thus enables to compare all five horizons within one test that naturally takes into account the dependence structure of horizons within one site. Finally the adjustment for multiple comparisons was done by randomly permuting the 5-dimensional response of the individual sites. For each random permutation the p-values of all the considered test were recalculated and minimum of these p-values was recorded. The distribution of those minimal p-values (created by random permutations) then yielded the needed hypothetical distribution for assessing statistical significance after a correction for multiple comparisons. Finally a linear mixed model [48] was used to assess the possible different effect of bacterial and fungal abundance on carbon transformation (e.g. DOC, SOC, enzymatic activities, organic acid concentrations). This model took the carbon transformation as a response. The abundance and the forest type (spruce and beech) were taken as fixed effects. These regressors were taken both separately as well as in the interaction. Then the statistical significance of this interaction term proves that the effect of abundance on the carbon transformation in the spruce forest is different from the corresponding effect in the beech forest.

Similarly as for the Hotteling's test the individual site were considered as independent replicates but the horizons within one site were not considered independent. This dependence was modelled by the random effect shared within one site. The adjustment for the multiple comparison was done in the same way as above by randomly permuting (relabelling) the sites.

From the model assumptions it is important that the homoscedasticity assumption is satisfied and distribution of the response is not too much different from a normal distribution (note that the perfect normality is not necessary thanks to the central limit theorem). The above model assumptions were checked visually as due to limited sample sizes formal test procedures would detect only very dramatic violations of homoscedasticity. The results of the diagnostic plots were satisfactory. This was definitely thanks to the fact that before any testing we checked the distribution of each of the analysed variables and log-transformed where it seems to be reasonable (to bring the distribution closer to a normal distribution).

Please keep the conclusions cautious:

Because the study was done at one locality and the forest type was not replicated, some conclusions should be clearly limited to this study site. Please avoid broad general statements about tree species effects, forest management, water retention, or ecosystem resilience unless they are directly supported by the data.

Please reduce speculative wording:

Some interpretations related to secondary metabolites, microbial competition, and antagonistic mechanisms should be written more cautiously. Please use wording such as “may indicate”, “suggests”, or “is consistent with”, where appropriate.

Yes, final check up was done and more cautious wording was applied and speculations removed.
