# Supplementary material for: Tree species influence soil carbon quality but not total storage across horizons: European beech on Dystric Cambisol and Norway spruce on Entic Podzol
Source: PLoS One. 2026 Jun 8;21(6):e0350656. doi: 10.1371/journal.pone.0350656 (PMC13245778; doi:10.1371/journal.pone.0350656)
Supplement: Peer review history

# Peer review: Decision Letter 0

**DOI:** 10.1371/journal.pone.0350656.r001

**Authors:** Michal Bosela

27 Feb 2026

PONE-D-26-03344

Tree species influence carbon quality but not total storage across soil horizons: a comparison of European beech and Norway spruce

PLOS One

Dear Dr. Mareckova,

Thank you for submitting your manuscript to PLOS ONE. After careful consideration, we feel that it has merit but does not fully meet PLOS ONE’s publication criteria as it currently stands. Therefore, we invite you to submit a revised version of the manuscript that addresses the points raised during the review process.

==============================

**ACADEMIC EDITOR:**

The manuscript is methodologically rich and contains valuable data. However, due to concerns related to statistical testing strategy, independence of samples, interpretation of functional genes, and confounding of tree species and soil type, I recommend a major revision.

The revised manuscript should:

- Clarify experimental design and replication structure

- Strengthen and possibly simplify statistical analyses

- Address multiple testing and model assumptions

- Moderate speculative interpretations

- Improve integration between microbial and SOC quality data

==============================

Please submit your revised manuscript by Apr 13 2026 11:59PM. If you will need more time than this to complete your revisions, please reply to this message or contact the journal office at plosone@plos.org. When you're ready to submit your revision, log on to https://www.editorialmanager.com/pone/ and select the 'Submissions Needing Revision' folder to locate your manuscript file.

Please include the following items when submitting your revised manuscript:

- A letter that responds to each point raised by the academic editor and reviewer(s). You should upload this letter as a separate file labeled 'Response to Reviewers'.
- A marked-up copy of your manuscript that highlights changes made to the original version. You should upload this as a separate file labeled 'Revised Manuscript with Track Changes'.
- An unmarked version of your revised paper without tracked changes. You should upload this as a separate file labeled 'Manuscript'.

If you would like to make changes to your financial disclosure, please include your updated statement in your cover letter. Guidelines for resubmitting your figure files are available below the reviewer comments at the end of this letter.

If applicable, we recommend that you deposit your laboratory protocols in protocols.io to enhance the reproducibility of your results. Protocols.io assigns your protocol its own identifier (DOI) so that it can be cited independently in the future. For instructions see: https://journals.plos.org/plosone/s/submission-guidelines#loc-laboratory-protocols. Additionally, PLOS ONE offers an option for publishing peer-reviewed Lab Protocol articles, which describe protocols hosted on protocols.io. Read more information on sharing protocols at https://plos.org/protocols?utm_medium=editorial-email&utm_source=authorletters&utm_campaign=protocols.

We look forward to receiving your revised manuscript.

Kind regards,

Michal Bosela, Ph.D.

Academic Editor

PLOS One

Journal Requirements:

When submitting your revision, we need you to address these additional requirements.

1. Please ensure that your manuscript meets PLOS ONE's style requirements, including those for file naming. The PLOS ONE style templates can be found at

https://journals.plos.org/plosone/s/file?id=wjVg/PLOSOne_formatting_sample_main_body.pdf and https://journals.plos.org/plosone/s/file?id=ba62/PLOSOne_formatting_sample_title_authors_affiliations.pdf

2. PLOS requires an ORCID iD for the corresponding author in Editorial Manager on papers submitted after December 6th, 2016. Please ensure that you have an ORCID iD and that it is validated in Editorial Manager. To do this, go to ‘Update my Information’ (in the upper left-hand corner of the main menu), and click on the Fetch/Validate link next to the ORCID field. This will take you to the ORCID site and allow you to create a new iD or authenticate a pre-existing iD in Editorial Manager.

3. In your Methods section, please provide additional information regarding the permits you obtained for the work. Please ensure you have included the full name of the authority that approved the field site access and, if no permits were required, a brief statement explaining why.

4. Please note that PLOS One has specific guidelines on code sharing for submissions in which author-generated code underpins the findings in the manuscript. In these cases, we expect all author-generated code to be made available without restrictions upon publication of the work. Please review our guidelines at https://journals.plos.org/plosone/s/materials-and-software-sharing#loc-sharing-code and ensure that your code is shared in a way that follows best practice and facilitates reproducibility and reuse.

5. Please include your full ethics statement in the ‘Methods’ section of your manuscript file. In your statement, please include the full name of the IRB or ethics committee who approved or waived your study, as well as whether or not you obtained informed written or verbal consent. If consent was waived for your study, please include this information in your statement as well.

6. Please provide a complete Data Availability Statement in the submission form, ensuring you include all necessary access information or a reason for why you are unable to make your data freely accessible. If your research concerns only data provided within your submission, please write "All data are in the manuscript and/or supporting information files" as your Data Availability Statement.

7. We note that the grant information you provided in the ‘Funding Information’ and ‘Financial Disclosure’ sections do not match.

When you resubmit, please ensure that you provide the correct grant numbers for the awards you received for your study in the ‘Funding Information’ section.

8. Thank you for stating the following financial disclosure:

The funding was obtained from the Technology Agency of the Czech Republic project SS05010039 and Ministry of Agriculture of the Czech Republic, Institutional support MZE-RO0425. The project was also supported by COST Action CA21138 - CLEANFOREST.

Please state what role the funders took in the study. If the funders had no role, please state: "The funders had no role in study design, data collection and analysis, decision to publish, or preparation of the manuscript."

If this statement is not correct you must amend it as needed.

Please include this amended Role of Funder statement in your cover letter; we will change the online submission form on your behalf.

9. Please amend your authorship list in your manuscript file to include author Marketa Mareckova.

10. Please amend the manuscript submission data (via Edit Submission) to include author Markéta Ságová-Marečková.

11. We note that Figure S1 in your submission contain [map/satellite] images which may be copyrighted. All PLOS content is published under the Creative Commons Attribution License (CC BY 4.0), which means that the manuscript, images, and Supporting Information files will be freely available online, and any third party is permitted to access, download, copy, distribute, and use these materials in any way, even commercially, with proper attribution. For these reasons, we cannot publish previously copyrighted maps or satellite images created using proprietary data, such as Google software (Google Maps, Street View, and Earth). For more information, see our copyright guidelines: http://journals.plos.org/plosone/s/licenses-and-copyright.

We require you to either (1) present written permission from the copyright holder to publish these figures specifically under the CC BY 4.0 license, or (2) remove the figures from your submission:

a. You may seek permission from the original copyright holder of Figure S1 to publish the content specifically under the CC BY 4.0 license.

We recommend that you contact the original copyright holder with the Content Permission Form (http://journals.plos.org/plosone/s/file?id=7c09/content-permission-form.pdf) and the following text:

“I request permission for the open-access journal PLOS ONE to publish XXX under the Creative Commons Attribution License (CCAL) CC BY 4.0 (http://creativecommons.org/licenses/by/4.0/). Please be aware that this license allows unrestricted use and distribution, even commercially, by third parties. Please reply and provide explicit written permission to publish XXX under a CC BY license and complete the attached form.”

Please upload the completed Content Permission Form or other proof of granted permissions as an "Other" file with your submission.

In the figure caption of the copyrighted figure, please include the following text: “Reprinted from [ref] under a CC BY license, with permission from [name of publisher], original copyright [original copyright year].”

b. If you are unable to obtain permission from the original copyright holder to publish these figures under the CC BY 4.0 license or if the copyright holder’s requirements are incompatible with the CC BY 4.0 license, please either i) remove the figure or ii) supply a replacement figure that complies with the CC BY 4.0 license. Please check copyright information on all replacement figures and update the figure caption with source information. If applicable, please specify in the figure caption text when a figure is similar but not identical to the original image and is therefore for illustrative purposes only.

The following resources for replacing copyrighted map figures may be helpful:

USGS National Map Viewer (public domain): http://viewer.nationalmap.gov/viewer/

The Gateway to Astronaut Photography of Earth (public domain): http://eol.jsc.nasa.gov/sseop/clickmap/

Maps at the CIA (public domain): https://www.cia.gov/library/publications/the-world-factbook/index.html and https://www.cia.gov/library/publications/cia-maps-publications/index.html

NASA Earth Observatory (public domain): http://earthobservatory.nasa.gov/

Landsat: http://landsat.visibleearth.nasa.gov/

USGS EROS (Earth Resources Observatory and Science (EROS) Center) (public domain): http://eros.usgs.gov/#

Natural Earth (public domain): http://www.naturalearthdata.com/

12. We note that Figure(s) 1, S1 in your submission contain copyrighted images. All PLOS content is published under the Creative Commons Attribution License (CC BY 4.0), which means that the manuscript, images, and Supporting Information files will be freely available online, and any third party is permitted to access, download, copy, distribute, and use these materials in any way, even commercially, with proper attribution. For more information, see our copyright guidelines: http://journals.plos.org/plosone/s/licenses-and-copyright.

We require you to either (1) present written permission from the copyright holder to publish these figures specifically under the CC BY 4.0 license, or (2) remove the figures from your submission:

a. You may seek permission from the original copyright holder of Figure(s) 1, S1 to publish the content specifically under the CC BY 4.0 license.

We recommend that you contact the original copyright holder with the Content Permission Form (http://journals.plos.org/plosone/s/file?id=7c09/content-permission-form.pdf) and the following text:

“I request permission for the open-access journal PLOS ONE to publish XXX under the Creative Commons Attribution License (CCAL) CC BY 4.0 (http://creativecommons.org/licenses/by/4.0/). Please be aware that this license allows unrestricted use and distribution, even commercially, by third parties. Please reply and provide explicit written permission to publish XXX under a CC BY license and complete the attached form.”

Please upload the completed Content Permission Form or other proof of granted permissions as an "Other" file with your submission.

In the figure caption of the copyrighted figure, please include the following text: “Reprinted from [ref] under a CC BY license, with permission from [name of publisher], original copyright [original copyright year].”

b. If you are unable to obtain permission from the original copyright holder to publish these figures under the CC BY 4.0 license or if the copyright holder’s requirements are incompatible with the CC BY 4.0 license, please either i) remove the figure or ii) supply a replacement figure that complies with the CC BY 4.0 license. Please check copyright information on all replacement figures and update the figure caption with source information. If applicable, please specify in the figure caption text when a figure is similar but not identical to the original image and is therefore for illustrative purposes only.

13. Please include captions for your Supporting Information files at the end of your manuscript, and update any in-text citations to match accordingly. Please see our Supporting Information guidelines for more information: http://journals.plos.org/plosone/s/supporting-information.

14. If the reviewer comments include a recommendation to cite specific previously published works, please review and evaluate these publications to determine whether they are relevant and should be cited. There is no requirement to cite these works unless the editor has indicated otherwise.

Additional Editor Comments :

The manuscript presents a detailed comparison of soil carbon distribution, organic matter quality, microbial community structure, and enzyme activities across complete soil profiles under European beech and Norway spruce at one study site. The study combines several analytical methods (soil chemistry, DRIFT spectroscopy, amplicon sequencing, ddPCR, enzyme measurements), which makes the dataset extensive and technically strong.

The main result, that total SOC stocks are similar between the two forest types, but carbon quality, vertical distribution, and microbial characteristics differ, is relevant for forest soil ecology and carbon cycling research. The inclusion of the full soil profile is an important strength of the study.

However, there are several issues that need to be addressed before publication:

1) The comparison is between two different soil types (Cambisol and Podzol), therefore the effects of tree species are partly confounded with soil type. In several parts, the manuscript attributes differences mainly to tree species without sufficiently discussing pedogenic effects.

2) Hypotheses are vague and not formally tested. Your three research questions are broad and exploratory.

3) The research was conducted at a single locality, which limits generalization of the conclusions. Some statements are formulated too broadly.

4) Some interpretations, especially regarding secondary metabolites and microbial competition, are rather speculative and should be presented more cautiously.

5) The Discussion is relatively long and descriptive, and would benefit from a clearer structure and a stronger synthesis of the main findings.

6) The manuscript relies on multiple Wilcoxon–Mann–Whitney tests applied separately for each horizon and variable which may raise some concerns.

Considering the quality of the data and the relevance of the topic, I recommend major revision. The revised version should:

1) More clearly distinguish tree species effects from soil-type effects

2) Rewrite aims as explicit hypotheses

3) Moderate causal and general conclusions

4) Improve hypothesis-driven interpretation

5) Shorten and better structure the Discussion

6) Using Wilcoxon–Mann–Whitney tests applied separately for each horizon and variable may raise concerns about:

- Multiple testing inflation (Type I error)

- Fragmented interpretation

- Lack of integrated multivariate analysis

The authors should:

- Indicate whether any correction for multiple comparisons (e.g. FDR) was applied.

- Consider multivariate approaches (e.g. PERMANOVA for chemistry, dbRDA linking SOC indices to microbial composition).

- Clarify the structure of the linear mixed models: What exactly was treated as random effect? Were horizons treated as nested within plots? Were assumptions of normality and homoscedasticity checked?

The mixed-model results are currently presented briefly and need clearer explanation of model structure, fixed effects, and interaction terms.

In addition to Methods:

Seven sampling sites per forest type are mentioned. It should be clearly stated:

- Whether horizons within one soil pit were treated as independent samples in statistical tests.

- How spatial independence between pits was ensured.

- Whether forest type is replicated at the landscape level (it appears not).

The manuscript should clarify the true level of replication to avoid pseudoreplication.

[Note: HTML markup is below. Please do not edit.]

Reviewers' comments:

Reviewer's Responses to Questions

**Comments to the Author**

1. Is the manuscript technically sound, and do the data support the conclusions?

The manuscript must describe a technically sound piece of scientific research with data that supports the conclusions. Experiments must have been conducted rigorously, with appropriate controls, replication, and sample sizes. The conclusions must be drawn appropriately based on the data presented.

Reviewer #1: Yes

**********

2. Has the statistical analysis been performed appropriately and rigorously?

Reviewer #1: Yes

**********

3. Have the authors made all data underlying the findings in their manuscript fully available?

The PLOS Data policy requires authors to make all data underlying the findings described in their manuscript fully available without restriction, with rare exception (please refer to the Data Availability Statement in the manuscript PDF file). The data should be provided as part of the manuscript or its supporting information, or deposited to a public repository. For example, in addition to summary statistics, the data points behind means, medians and variance measures should be available. If there are restrictions on publicly sharing data—e.g. participant privacy or use of data from a third party—those must be specified.

Reviewer #1: Yes

**********

4. Is the manuscript presented in an intelligible fashion and written in standard English?

PLOS ONE does not copyedit accepted manuscripts, so the language in submitted articles must be clear, correct, and unambiguous. Any typographical or grammatical errors should be corrected at revision, so please note any specific errors here.

Reviewer #1: Yes

**********

5. Review Comments to the Author

Please use the space provided to explain your answers to the questions above. You may also include additional comments for the author, including concerns about dual publication, research ethics, or publication ethics. (Please upload your review as an attachment if it exceeds 20,000 characters)

Reviewer #1: Dear Authors,

Thank you for the interesting manuscript. The submitted manuscript presents important findings that elucidate the differing effects of deciduous and coniferous litter on selected soil properties.

All my comments, questions, and suggestions are provided in the attached file.

Kind regards,

Reviewer

**********

6. PLOS authors have the option to publish the peer review history of their article (what does this mean?). If published, this will include your full peer review and any attached files.

If you choose “no”, your identity will remain anonymous but your review may still be made public.

**Do you want your identity to be public for this peer review?** For information about this choice, including consent withdrawal, please see our Privacy Policy.

Reviewer #1: No

**********

[NOTE: If reviewer comments were submitted as an attachment file, they will be attached to this email and accessible via the submission site. Please log into your account, locate the manuscript record, and check for the action link "View Attachments". If this link does not appear, there are no attachment files.]

To ensure your figures meet our technical requirements, please review our figure guidelines: https://journals.plos.org/plosone/s/figures

You may also use PLOS’s free figure tool, NAAS, to help you prepare publication quality figures: https://journals.plos.org/plosone/s/figures#loc-tools-for-figure-preparation.

NAAS will assess whether your figures meet our technical requirements by comparing each figure against our figure specifications.

Attachment Submitted filename: review_of_PONE-D-26-03344.pdf

# Author comment: Author response to Decision Letter 1

**DOI:** 10.1371/journal.pone.0350656.r002

30 Apr 2026

Editor

1) The comparison is between two different soil types (Cambisol and Podzol), therefore the effects of tree species are partly confounded with soil type. In several parts, the manuscript attributes differences mainly to tree species without sufficiently discussing pedogenic effects.

A:Thank you for the comment. The relationship between both tree species and soil type was now elaborated in several parts of the MS including the title.

2) Hypotheses are vague and not formally tested. Your three research questions are broad and exploratory.

A:The hypotheses were changed so they would correspond to the tested relationships.

L114-124

3) The research was conducted at a single locality, which limits generalization of the conclusions. Some statements are formulated too broadly.

A:The statements and explanations were modified throughout the MS.

4) Some interpretations, especially regarding secondary metabolites and microbial competition, are rather speculative and should be presented more cautiously.

A:This part of MS in Discussion was shortened and speculations removed. Some information on why antibiotics and secondary metabolites are important to study together with decomposition processes was added to the introduction.

L 85-96

5) The Discussion is relatively long and descriptive, and would benefit from a clearer structure and a stronger synthesis of the main findings.

A:The discussion was substantially shortened from 1904 words to 1148 words.

6) The manuscript relies on multiple Wilcoxon–Mann–Whitney tests applied separately for each horizon and variable which may raise some concerns.

A:The Wilcoxon–Mann–Whitney tests were replaced by Hotteling's two sample test and corrections for multiple testing was done by permutation. This method was recommended by our colleague Marek Omelka Ph.D., who teaches statistics at Charles University and does consulting for environmental and ecological studies.

6) Using Wilcoxon–Mann–Whitney tests applied separately for each horizon and variable may raise concerns about:

- Multiple testing inflation (Type I error)

- Fragmented interpretation

- Lack of integrated multivariate analysis

A:Yes, we understand this problem. In the original paper we omitted the Type I error corrections because we did not know how to handle the strong differences between the individual horizons, which in fact were not the focus of the paper as we know that horizons in soil profiles differ. Thus, it seemed that testing for those differences is meaningless. However, our upon your request and with help of our statistician the testing was modified.

See methods.

L 287-291

The authors should:

- Indicate whether any correction for multiple comparisons (e.g. FDR) was applied.

- Consider multivariate approaches (e.g. PERMANOVA for chemistry, dbRDA linking SOC indices to microbial composition).

A:Yes. As mentioned above the multiple testing was assessed by permanova now.

- Clarify the structure of the linear mixed models: What exactly was treated as random effect? Were horizons treated as nested within plots? Were assumptions of normality and homoscedasticity checked? The mixed-model results are currently presented briefly and need clearer explanation of model structure, fixed effects, and interaction terms.

Yes, the horizons were treated as nested within plots. The logarithmic transformation was applied were it was useful to bring the data closer to normality and heteroscedasticity assumptions. These assumptions were also checked and seemed to be satisfied in a reasonable way.

A:Linear mixed-effects models were used with the measurements from the same

site e.g. B1L, B1F, B1H B1A, B1B, ,sharing the same random effect. These random effects enable the model to account for the variability that comes from the possible dependence of the measurements from the same location.

Reference: Venables, WN, and Ripley BD. Modern Applied Statistics with

S. Springer, New York, NY, 2002.

In addition to Methods:

Seven sampling sites per forest type are mentioned. It should be clearly stated:

- Whether horizons within one soil pit were treated as independent samples in statistical tests.

- How spatial independence between pits was ensured.

Spatial independence was retained by selecting sites with each forest, which were not connected by surface or subsurface water flow, they were about

- Whether forest type is replicated at the landscape level (it appears not).

A: The sampling was re-written to address these issues. They were further addressed in statistical models.

The manuscript should clarify the true level of replication to avoid pseudoreplication.

A: While soil pits can be considered as independent replicates the horizons within one soil pit cannot be treated as independent samples. But this is very naturally incorporated in the used the Hotelling's two sample test which takes the measurements at different horizons within one soil split as a multivariate (5-dimensional) response.

[Note: HTML markup is below. Please do not edit.]

Reviewer:

Major comments

I believe that mycorrhizae could also play a crucial role in the acquisition of water and nutrients from the soil, particularly under conditions of low nutrient availability or in acidic soil environments. This represents another important factor that could play a significant role. Especially for tree nutrition.

A: Yes, we agree. However, we did not study mycorrhiza in this work, so we cannot give more specific comments on this issue. Some connections to our results are done by citing literature e.g. the study by Uroz et al. 2016. E.g. L 436,

Another important factor could be the chemistry of the mineral soil, particularly within the rooting zone. This is also documented in the manuscript. However, I did not find any information about the depth of mineral soil (B horizon) sampling.

A: The sampling went 25 cm deep in B horizon under both trees. It was not possible to dig deeper because larger stones started to appear. The situation was similar under both trees, so there was not any difference in B horizon thickness.

The information is now on

L 163.

Overall, the text is well written and clearly explains the background and methodology. The results are well presented and easy to understand. The authors formulated three research questions in the Introduction; therefore, readers might expect explicit answers to these questions at the end of the Discussion or within the Conclusion section, summarizing whether and how the study addressed them.

A: Yes, the answers are now more clearly replied in both Discussion and Conclusions.

Minor comments

I have the following minor comments/questions on the paper:

References are numbered throughout the text; however, in line 54, “Augusto et al. 2002” is cited in text format. Please delete this citation style or renumber the reference list accordingly.

Done.

Pages 99–114: I suggest moving this section to the Materials and Methods. This part describes the methodological procedure. The Introduction could instead conclude with the research questions.

A: Yes, thank you. We shortened this part only to give a brief methodological introduction to the reader and finished the Introduction with hypotheses. Longer methodological description is provided in Methods.

L 105-124

Section 2.2 Soil sampling: There is no information about the depth of B horizon sampling. How deep were the B horizon samples taken? Alternatively, how thick was the subsoil layer from which the samples were collected? Additionally, regarding rock fragment content – you mention that “all sites were about three meters from the closest tree and on a homogeneous substrate (without larger stones).” Does this mean that larger stones were present at the site, possibly not only on the surface but also within the soil profile? What was the proportion of rock fragments?

A: The depth of B horizon was about 25 cm, where more skeletal structure occurred. However, on the surface and in the upper horizons there were no stones in the soil. The stones mentioned are rather small rocks, which are randomly dispseudotributed at the site. This was explained in the methods more carefully.

L 146-148

- I did not check the completeness of the reference list nor cross-check the references cited in the text. Please review this carefully to avoid inconsistencies.

Yes, that was done for this new shorter version.

Attachment Submitted filename: responsereviewers.docx

# Editor report: Decision Letter 1

**DOI:** 10.1371/journal.pone.0350656.r003

**Authors:** Michal Bosela

8 May 2026

PONE-D-26-03344R1

Tree species influence carbon quality but not total storage across soil horizons: a comparison of European beech and Norway spruce

PLOS One

Dear Dr. Mareckova,

Thank you for submitting your manuscript to PLOS ONE. After careful consideration, we feel that it has merit but does not fully meet PLOS ONE’s publication criteria as it currently stands. Therefore, we invite you to submit a revised version of the manuscript that addresses the points raised during the review process.

Please submit your revised manuscript by Jun 22 2026 11:59PM. If you will need more time than this to complete your revisions, please reply to this message or contact the journal office at plosone@plos.org. When you're ready to submit your revision, log on to https://www.editorialmanager.com/pone/ and select the 'Submissions Needing Revision' folder to locate your manuscript file.

Please include the following items when submitting your revised manuscript:

- A letter that responds to each point raised by the academic editor and reviewer(s). You should upload this letter as a separate file labeled 'Response to Reviewers'.
- A marked-up copy of your manuscript that highlights changes made to the original version. You should upload this as a separate file labeled 'Revised Manuscript with Track Changes'.
- An unmarked version of your revised paper without tracked changes. You should upload this as a separate file labeled 'Manuscript'.

If you would like to make changes to your financial disclosure, please include your updated statement in your cover letter. Guidelines for resubmitting your figure files are available below the reviewer comments at the end of this letter.

If applicable, we recommend that you deposit your laboratory protocols in protocols.io to enhance the reproducibility of your results. Protocols.io assigns your protocol its own identifier (DOI) so that it can be cited independently in the future. For instructions see: https://journals.plos.org/plosone/s/submission-guidelines#loc-laboratory-protocols. Additionally, PLOS ONE offers an option for publishing peer-reviewed Lab Protocol articles, which describe protocols hosted on protocols.io. Read more information on sharing protocols at https://plos.org/protocols?utm_medium=editorial-email&utm_source=authorletters&utm_campaign=protocols.

As the corresponding author, your ORCID iD is verified in the submission system and will appear in the published article. PLOS supports the use of ORCID, and we encourage all coauthors to register for an ORCID iD and use it as well. Please encourage your coauthors to verify their ORCID iD within the submission system before final acceptance, as unverified ORCID iDs will not appear in the published article. *Only* the individual author can complete the verification step; PLOS staff *cannot* verify ORCID iDs on behalf of authors.

We look forward to receiving your revised manuscript.

Kind regards,

Michal Bosela, Ph.D.

Academic Editor

PLOS One

Journal Requirements:

If the reviewer comments include a recommendation to cite specific previously published works, please review and evaluate these publications to determine whether they are relevant and should be cited. There is no requirement to cite these works unless the editor has indicated otherwise.

Please review your reference list to ensure that it is complete and correct. If you have cited papers that have been retracted, please include the rationale for doing so in the manuscript text, or remove these references and replace them with relevant current references. Any changes to the reference list should be mentioned in the rebuttal letter that accompanies your revised manuscript. If you need to cite a retracted article, indicate the article’s retracted status in the References list and also include a citation and full reference for the retraction notice.

**Additional Editor Comments:**

The revision improved the manuscript substantially. In particular, the manuscript now better acknowledges that the comparison is between beech on Dystric Cambisol and spruce on Entic Podzol, and not only between two tree species. The hypotheses are clearer, the single-site design is described more openly, the B horizon sampling depth is now specified, and the Discussion is shorter and more focused.

However, before the manuscript can be accepted, I would like to ask you for a further minor revision. Please address the following points carefully:

Statistical methods:

Please describe more precisely how the Hotelling’s two-sample test was applied, how the permutation correction for multiple testing was done, and which tests were corrected. Also, please make the terminology consistent. In the response letter you mention PERMANOVA, but in the manuscript the methods refer mainly to Hotelling’s test and AMOVA.

Linear mixed models:

Please clearly describe the model structure: what was the response variable, what were the fixed effects, what was the random effect, and how horizons and sampling sites were treated. Please also briefly state how model assumptions were checked.

Please keep the conclusions cautious:

Because the study was done at one locality and the forest type was not replicated, some conclusions should be clearly limited to this study site. Please avoid broad general statements about tree species effects, forest management, water retention, or ecosystem resilience unless they are directly supported by the data.

Please reduce speculative wording:

Some interpretations related to secondary metabolites, microbial competition, and antagonistic mechanisms should be written more cautiously. Please use wording such as “may indicate”, “suggests”, or “is consistent with”, where appropriate.

In my view, the manuscript is close to being acceptable, but these points should be corrected before final acceptance.

[Note: HTML markup is below. Please do not edit.]

[NOTE: If reviewer comments were submitted as an attachment file, they will be attached to this email and accessible via the submission site. Please log into your account, locate the manuscript record, and check for the action link "View Attachments". If this link does not appear, there are no attachment files.]

To ensure your figures meet our technical requirements, please review our figure guidelines: https://journals.plos.org/plosone/s/figures

You may also use PLOS’s free figure tool, NAAS, to help you prepare publication quality figures: https://journals.plos.org/plosone/s/figures#loc-tools-for-figure-preparation.

NAAS will assess whether your figures meet our technical requirements by comparing each figure against our figure specifications.

# Author comment: Author response to Decision Letter 2

**DOI:** 10.1371/journal.pone.0350656.r004

13 May 2026

Additional Editor Comments:

Statistical methods:

Please describe more precisely how the Hotelling’s two-sample test was applied, how the permutation correction for multiple testing was done, and which tests were corrected. Also, please make the terminology consistent. In the response letter you mention PERMANOVA, but in the manuscript the methods refer mainly to Hotelling’s test and AMOVA.

Linear mixed models:

Please clearly describe the model structure: what was the response variable, what were the fixed effects, what was the random effect, and how horizons and sampling sites were treated. Please also briefly state how model assumptions were checked.

Yes, see below an explanation by our statistician Marek Omelka.

Statistical differences of soil variables and enzymatic activities were assessed by Hotelling‘s two-sample test [47]. The individual site were considered independent replicates, while the horizons within one site were treated as a multivariate (5-dimensional) response. The test then compares the multivariate means (each component of the mean corresponding to one horizon) between two samples representing spruce and beech forests. The test thus enables to compare all five horizons within one test that naturally takes into account the dependence structure of horizons within one site. Finally the adjustment for multiple comparisons was done by randomly permuting the 5-dimensional response of the individual sites. For each random permutation the p-values of all the considered test were recalculated and minimum of these p-values was recorded. The distribution of those minimal p-values (created by random permutations) then yielded the needed hypothetical distribution for assessing statistical significance after a correction for multiple comparisons. Finally a linear mixed model [48] was used to assess the possible different effect of bacterial and fungal abundance on carbon transformation (e.g. DOC, SOC, enzymatic activities, organic acid concentrations). This model took the carbon transformation as a response. The abundance and the forest type (spruce and beech) were taken as fixed effects. These regressors were taken both separately as well as in the interaction. Then the statistical significance of this interaction term proves that the effect of abundance on the carbon transformation in the spruce forest is different from the corresponding effect in the beech forest.

Similarly as for the Hotteling's test the individual site were considered as independent replicates but the horizons within one site were not considered independent. This dependence was modelled by the random effect shared within one site. The adjustment for the multiple comparison was done in the same way as above by randomly permuting (relabelling) the sites.

From the model assumptions it is important that the homoscedasticity assumption is satisfied and distribution of the response is not too much different from a normal distribution (note that the perfect normality is not necessary thanks to the central limit theorem). The above model assumptions were checked visually as due to limited sample sizes formal test procedures would detect only very dramatic violations of homoscedasticity. The results of the diagnostic plots were satisfactory. This was definitely thanks to the fact that before any testing we checked the distribution of each of the analysed variables and log-transformed where it seems to be reasonable (to bring the distribution closer to a normal distribution).

Please keep the conclusions cautious:

Because the study was done at one locality and the forest type was not replicated, some conclusions should be clearly limited to this study site. Please avoid broad general statements about tree species effects, forest management, water retention, or ecosystem resilience unless they are directly supported by the data.

Please reduce speculative wording:

Some interpretations related to secondary metabolites, microbial competition, and antagonistic mechanisms should be written more cautiously. Please use wording such as “may indicate”, “suggests”, or “is consistent with”, where appropriate.

Yes, final check up was done and more cautious wording was applied and speculations removed.

Attachment Submitted filename: Response to Reviewers_0511.docx

# Editor report: Decision Letter 2

**DOI:** 10.1371/journal.pone.0350656.r005

**Authors:** Michal Bosela

17 May 2026

Tree species influence soil carbon quality but not total storage across horizons: European beech on Dystric Cambisol and Norway spruce on Entic Podzol

PONE-D-26-03344R2

Dear Dr. Mareckova,

We’re pleased to inform you that your manuscript has been judged scientifically suitable for publication and will be formally accepted for publication once it meets all outstanding technical requirements.

Within one week, you’ll receive an e-mail detailing the required amendments. When these have been addressed, you’ll receive a formal acceptance letter and your manuscript will be scheduled for publication.

An invoice will be generated when your article is formally accepted. Please note, if your institution has a publishing partnership with PLOS and your article meets the relevant criteria, all or part of your publication costs will be covered. Please make sure your user information is up-to-date by logging into Editorial Manager at Editorial Manager® and clicking the ‘Update My Information' link at the top of the page. For questions related to billing, please contact billing support.

If your institution or institutions have a press office, please notify them about your upcoming paper to help maximize its impact. If they’ll be preparing press materials, please inform our press team as soon as possible -- no later than 48 hours after receiving the formal acceptance. Your manuscript will remain under strict press embargo until 2 pm Eastern Time on the date of publication. For more information, please contact onepress@plos.org.

Kind regards,

Michal Bosela, Ph.D.

Academic Editor

PLOS One

Additional Editor Comments (optional):

Reviewers' comments:

# Editor report: Acceptance letter

**DOI:** 10.1371/journal.pone.0350656.r006

**Authors:** Michal Bosela

PONE-D-26-03344R2

PLOS One

Dear Dr. Mareckova,

I'm pleased to inform you that your manuscript has been deemed suitable for publication in PLOS One. Congratulations! Your manuscript is now being handed over to our production team.

At this stage, our production department will prepare your paper for publication. This includes ensuring the following:

* All references, tables, and figures are properly cited

* All relevant supporting information is included in the manuscript submission,

* There are no issues that prevent the paper from being properly typeset

You will receive further instructions from the production team, including instructions on how to review your proof when it is ready. Please keep in mind that we are working through a large volume of accepted articles, so please give us a few days to review your paper and let you know the next and final steps.

Lastly, if your institution or institutions have a press office, please let them know about your upcoming paper now to help maximize its impact. If they'll be preparing press materials, please inform our press team within the next 48 hours. Your manuscript will remain under strict press embargo until 2 pm Eastern Time on the date of publication. For more information, please contact onepress@plos.org.

You will receive an invoice from PLOS for your publication fee after your manuscript has reached the completed accept phase. If you receive an email requesting payment before acceptance or for any other service, this may be a phishing scheme. Learn how to identify phishing emails and protect your accounts at https://explore.plos.org/phishing.

If we can help with anything else, please email us at customercare@plos.org.

Thank you for submitting your work to PLOS ONE and supporting open access.

Kind regards,

PLOS ONE Editorial Office Staff

on behalf of

Dr. Michal Bosela

Academic Editor

PLOS One
